# Oral‐Rinse‐Sourced Microbiota in Oral Health and Diseases in a Representative US Adult Population: Implications for Diagnostics

**DOI:** 10.1111/jcpe.70101

**Published:** 2026-01-14

**Authors:** Yu Xie, Alejandro Artacho, Xiaoyu Yu, Mengning Bi, Hairui Li, Yuan Li, Andrea Roccuzzo, Alex Mira, Bob T. Rosier, Maurizio S. Tonetti

**Affiliations:** ^1^ Shanghai Perio‐Implant Innovation Center, Institute for Oral, Craniofacial and Sensory Research Ninth People's Hospital, Shanghai Jiao Tong University School of Medicine Shanghai China; ^2^ National Clinical Research Center of Oral Diseases and National Center of Stomatology, College of Stomatology, Shanghai Jiao Tong University School of Medicine Shanghai China; ^3^ Department of Genomics and Health FISABIO Foundation, Center for Advanced Research in Public Health Valencia Spain; ^4^ European Research Group on Periodontology (ERGOPerio) Genova Italy

**Keywords:** 16S rRNA, dental caries, diagnosis, machine learning, microbiota, periodontitis

## Abstract

**Aims:**

To investigate the associations between oral‐rinse microbiota and distinct oral conditions, and further evaluate its potential ability to distinguish periodontitis severity.

**Methods:**

Oral‐rinse‐sourced microbiota with 16S ribosomal RNA sequencing from 3770 adults in US National Health and Nutrition Examination Survey 2009–2012 were analysed across oral health, caries, periodontitis, co‐existing caries and periodontitis and edentulism. Diagnostic potential of the oral‐rinse microbiota for periodontitis severity was evaluated using multi‐class random forest (RF) model with internal validation and external validation in an independent cohort (*n* = 392).

**Results:**

Oral condition accounted for substantial variance in oral‐rinse microbiota, revealing disease or tooth loss–associated shifts. Increasing acidogenic/aciduric taxa (*Veillonella*, *Lactobacillus*, *Atopobium*) or periodontitis‐associated taxa (*Filifactor*, *Treponema*, *Tannerella*) were identified in caries‐only or periodontitis‐only groups, respectively, while the co‐existing disease group showed overlapping shifts. Taxa shifted dose‐dependently with increasing periodontitis severity. The RF model achieved moderate performance in identifying severe periodontitis, with the area under the receiver operating characteristic curve (AUROC) of 0.81 (0.75–0.87) internally and 0.83 (0.77–0.88) externally. Key contributing taxa aligned with established periodontitis‐associated genera, supporting model interpretability.

**Conclusion:**

Based on our results, oral‐rinse microbiota captures disease‐specific signatures across oral conditions, supporting its potential as a non‐invasive tool to monitor oral microbial ecology and assess periodontitis severity at the population level.

## 1 Introduction

The oral cavity is a microbially rich environment with multiple colonisation niches, including tooth surfaces, the tongue and the oral mucosa (Mark Welch et al. 2020). Microbial communities exhibit distinct composition profiles that are affected by local environmental conditions (Rosier et al. 2018). In oral health, the oral microbiota and the host maintain a dynamic equilibrium. However, ecological imbalances can lead to a shift towards a pathogenic state, either locally or throughout the mouth, especially for teeth, whose hard, non‐shedding surfaces support the formation and maturation of biofilms (Easter et al. 2023; Kaan et al. 2021). Dysbiosis of tooth‐associated microbial biofilms initiates both caries and periodontitis (Lamont et al. 2018; Sedghi et al. 2021). These oral diseases rank among the most prevalent non‐communicable diseases globally, impacting billions of individuals of all ages. They significantly contribute to the global burden of disease, accounting for a considerable loss of disability‐adjusted life years (DALYs) and are directly responsible for up to 10% of total medical expenses (GBD 2021 Oral Disorders Collaborators 2025). Beyond oral health, these oral diseases also affect nutritional intake and the quality of life, and increase the risk of systemic diseases (Hajishengallis and Chavakis 2021; Liu et al. 2024; Uy et al. 2022; Villoria et al. 2024).

Caries and periodontitis are driven by the microbiome localised on supragingival and subgingival tooth surfaces, with characteristic microbial shifts and site‐specific environmental changes (Feres et al. 2021; Marsh 1994; Takahashi and Nyvad 2011). Beyond site‐focused studies, it is equally important to investigate whether these disease‐related microbial alterations can be captured in oral fluids that integrate signals across the mouth, thereby distinguishing disease status. Oral‐rinse sampling aggregates microbiota from multiple intraoral niches, offering a composite snapshot of supragingival, subgingival and other oral environments (Katsiki et al. 2021). Given its non‐invasive and simple collection, oral rinse has the potential for population‐level surveillance and microbiome‐based diagnostics of oral diseases. Recent studies have indicated that oral‐rinse‐sourced microbiota are associated with demographic and socioeconomic factors (Chaturvedi et al. 2025; Qi et al. 2025). Yet, the relationship between oral‐rinse microbiota and oral health, considering caries and periodontitis simultaneously, remains underexplored.

The United States National Health and Nutrition Examination Survey (NHANES) is a nationally representative programme that employs a multistage probability sampling design to survey the non‐institutionalised US population (Centers for Disease Control and Prevention and Statistics 2024a). Since 1999, NHANES has offered continuous cross‐sectional data, including questionnaires, physical examinations and laboratory measurements. During the consecutive cycles of 2009–2010 and 2011–2012, oral rinse samples were collected and analysed by 16S rRNA gene amplicon sequencing, producing genus‐level microbial profiles for thousands of participants (Centers for Disease Control and Prevention and Statistics 2024b, 2024c). Although 16S rRNA sequencing offers only limited taxonomic resolution and read length, its application in oral rinse samples at this scale provides a unique opportunity to elucidate the links between the oral‐rinse‐sourced microbiota and its association with oral diseases.

Therefore, this study aims to profile the differences in microbiota sourced from oral rinses across various oral conditions, utilising data from the United States NHANES database. The main objective of this study is to characterise oral rinse microbiota profiles across the oral health spectrum, including oral health, dental caries alone, periodontitis alone, co‐existing caries and periodontitis and edentulism. Additionally, it aims to further assess the relationship between oral‐rinse‐sourced microbiota and the severity of periodontitis. Lastly, the study aims to explore key periodontitis‐associated taxa in the oral rinse and evaluate their potential diagnostic value for periodontitis through a machine learning approach.

## 2 Methods

### 2.1 Study Population

This study used two cross‐sectional cohorts: (i) the US NHANES cohort to characterise oral‐rinse microbiota, examine associations with distinct oral conditions and develop a microbiota‐based machine learning model for periodontitis diagnosis; and (ii) an investigator‐initiated clinical cohort for external validation of the machine learning model.

NHANES is a nationally representative, cross‐sectional programme employing a complex, multistage probability sampling design. Data are collected via standardised in‐home interviews and clinical examinations conducted at mobile examination centres (MECs). Detailed protocols are publicly available (https://www.cdc.gov/nchs/nhanes/index.htm). Participants from the 2009–2010 and 2011–2012 cycles with 16S rRNA sequencing data from oral‐rinse samples were included (Centers for Disease Control and Prevention and Statistics 2024b, 2024c). Individuals were excluded if they were < 18 years of age, pregnant, lacked dental examination, lacked either caries or periodontal records or had only one remaining tooth (precluding periodontal assessment) (Figure S1) (Eke et al. 2012; Holtfreter et al. 2024). The NHANES protocol was approved by the National Center for Health Statistics' Ethics Review Board.

The external‐validation cohort comprised consecutively recruited dentate adults seeking dental care at the Shanghai Ninth People's Hospital (July 2023–July 2024). Exclusions were pregnancy, antibiotic use within 3 months and professional periodontal treatment within 12 months. All participants provided an oral‐rinse sample and then underwent standardised periodontal examinations. The study was approved by the Research Ethics Committee of Shanghai Ninth People's Hospital (SH9H‐2021‐T408‐3).

### 2.2 Oral Health Status Definitions

Oral health was defined by dentition, dental caries and periodontitis status. Participants were defined as edentulous if all teeth were recorded as missing. Dentate individuals were further classified into four groups: (i) orally healthy (neither caries nor periodontitis present); (ii) caries only; (iii) periodontitis only; and (iv) co‐existing caries and periodontitis. Periodontitis was identified using the Centers for Disease Control and Prevention/American Academy of Periodontology (CDC/AAP) classification system and the Application of the 2018 Periodontal Status Classification to Epidemiologic data (ACES) classification system separately. Details of caries and periodontal examinations are provided in Supporting Information.

### 2.3 Oral‐Rinse Collection and 16S rRNA Gene Sequencing

In both cohorts, oral‐rinse samples were collected before dental examination. Trained examiners instructed participants to rinse and gargle with saline mouthwash/distilled water for 30 s. Samples were stored at −80°C before sequencing.

For 16S ribosomal RNA (16S rRNA) gene sequencing, the NHANES cohort targeted the V4 region using primers 515F and 806R, whereas the external‐validation cohort targeted V3–V4 using primers 341F and 806R. Amplicon data were processed with QIIME1 (v1.9.1, NHANES) or QIIME2 (v2025.4.0, external‐validation cohort), and taxonomy was assigned using the SILVA database (v123 for NHANES; v138.1 for external‐validation cohort). Additional sequencing and processing details are provided in Supporting Information and in previous publication (Centers for Disease Control and Prevention and Statistics 2025).

### 2.4 Statistical Analyses

Population characteristics were summarised and compared across different oral conditions. All microbial analyses and visualisations were conducted using the filtered dataset (Figure S1). Microbiota analyses included α‐diversity (Shannon, Chao1, Faith's PD), β‐diversity using Aitchison distances from centred log‐ratio (CLR)‐transformed data and the subgingival microbial dysbiosis index (SMDI; Chen et al. 2022). Dominant taxa were defined as those with average relative abundance of > 1% in each group. Associations between CLR‐transformed taxa and oral conditions were evaluated using MaAsLin2, adjusted for relevant covariates. Differential abundance between diseased groups and the orally healthy group was assessed using ALDEx2. Non‐compositional microbial analyses were conducted as sensitivity analyses.

For the machine learning pipeline, least absolute shrinkage and selection operator (LASSO) was applied for feature selection. Afterwards, a random forest (RF) classifier was trained to classify different periodontitis severity from CLR‐transformed data of oral‐rinse microbiota, with hyperparameters tuned via GridSearchCV using 10‐fold cross‐validation. The optimal model was internally validated using an independent held‐out testing set and externally validated in the independent investigator‐initiated cohort. Performance metrics included class‐wise area under the receiver operating characteristic curve (AUROC), accuracy, sensitivity and specificity with 95% confidence intervals (95% CIs). Model interpretability was assessed with Shapley Additive exPlanations (SHAP; Lundberg and Lee 2017).

Microbiome analyses in this study were conducted at the sample level without applying NHANES survey weights, to maintain internal consistency across downstream microbial analyses. All tests were two‐sided with α = 0.05. Analyses were performed in R (v4.4.1; R Core Team 2024) and Python (v3.12; Python Software Foundation 2023). Further details of statistical analyses and machine learning model development are provided in Supporting Information.

## 3 Results

### 3.1 Characteristics of Participants

A total of 3770 participants from NHANES with 77 genus‐level taxa were included for analyses. For balance across groups, analyses used the CDC/AAP definition in the main text (Figure S1), with ACES‐based results in Supporting Information. Of the 3770 participants, 293 were edentulous; among the dentate participants (*n* = 3477), 1056 had oral health, 440 caries only, 877 periodontitis only and 1104 co‐existing caries and periodontitis. Sociodemographic details under the CDC/AAP and ACES definition are shown in Tables 1 and S1.

**TABLE 1:** Characteristics of study participants with different oral health conditions based on caries and periodontitis status with CDC/AAP periodontitis definitions.

CDC/AAP definition	Overall	Edentulous	Oral health	Caries	Periodontitis	Caries + Periodontitis	*p*
*N*	3770	293	1056	440	877	1104	
Age	49 (39–59)	61 (54–65)	44 (36–54)	42 (35–50)	53 (44–62)	51 (41–59)	**< 0.001**
Sex							
Male	1955 (51.9%)	154 (52.6%)	417 (39.5%)	179 (40.7%)	501 (57.1%)	704 (63.8%)	**< 0.001**
Female	1815 (48.1%)	139 (47.4%)	639 (60.5%)	261 (59.3%)	376 (42.9%)	400 (36.2%)	
Race/ethnicity							
Mexican American	574 (15.2%)	18 (6.1%)	88 (8.3%)	71 (16.1%)	109 (12.4%)	288 (26.1%)	**< 0.001**
Other Hispanic	375 (9.9%)	33 (11.3%)	111 (10.5%)	34 (7.7%)	110 (12.5%)	87 (7.9%)	
Non‐Hispanic White	1305 (34.6%)	140 (47.8%)	458 (43.4%)	184 (41.8%)	225 (25.7%)	298 (27%)	
Non‐Hispanic Black	994 (26.4%)	79 (27%)	188 (17.8%)	121 (27.5%)	251 (28.6%)	355 (32.2%)	
Other race, including Multi‐racial	522 (13.8%)	23 (7.8%)	211 (20%)	30 (6.8%)	182 (20.8%)	76 (6.9%)	
BMI (kg/m^2^)	28.3 (24.7–32.9)	27.7 (24.5–33.1)	27.3 (24.0–31.8)	29.3 (25.4–33.8)	28.4 (25.0–32.9)	28.7 (25.0–33.4)	**< 0.001**
Income‐poverty ratio	1.8 (1.0–4.0)	1.1 (0.8–2.0)	3.6 (1.6–5.0)	1.6 (0.8–3.5)	2.0 (1.1–4.1)	1.2 (0.8–2.3)	**< 0.001**
Educational level							
Less than 9th grade	433 (11.5%)	56 (19.1%)	46 (4.4%)	32 (7.3%)	98 (11.2%)	201 (18.2%)	**< 0.001**
9–11th grade	632 (16.8%)	84 (28.7%)	86 (8.1%)	71 (16.1%)	117 (13.3%)	274 (24.8%)	
High school grad/GED or equivalent	834 (22.1%)	73 (24.9%)	140 (13.3%)	126 (28.6%)	213 (24.3%)	282 (25.5%)	
Some college or AA degree	1013 (26.9%)	64 (21.8%)	294 (27.8%)	147 (33.4%)	253 (28.8%)	255 (23.1%)	
College graduate or above	856 (22.7%)	16 (5.5%)	490 (46.4%)	63 (14.3%)	195 (22.2%)	92 (8.3%)	
Missing	2 (0.1%)	0 (0.0%)	0 (0.0%)	1 (0.2%)	1 (0.1%)	0 (0.0%)	
Alcohol consumption							
Never	687 (18.2%)	103 (35.2%)	112 (10.6%)	81 (18.4%)	155 (17.7%)	236 (21.4%)	**< 0.001**
Everyday/drink	158 (4.2%)	11 (3.8%)	25 (2.4%)	15 (3.4%)	44 (5%)	63 (5.7%)	
Every week/drink	930 (24.7%)	41 (14%)	309 (29.3%)	98 (22.3%)	214 (24.4%)	268 (24.3%)	
Every month/drink	532 (14.1%)	28 (9.6%)	192 (18.2%)	57 (13%)	117 (13.3%)	138 (12.5%)	
Several months/drink	677 (18%)	57 (19.5%)	193 (18.3%)	96 (21.8%)	162 (18.5%)	169 (15.3%)	
Missing	786 (20.8%)	53 (18.1%)	225 (21.3%)	93 (21.1%)	185 (21.1%)	230 (20.8%)	
Smoking status							
Never	1949 (51.7%)	65 (22.2%)	732 (69.3%)	233 (53.0%)	446 (50.9%)	473 (42.8%)	**< 0.001**
Former smoker	867 (23.0%)	94 (32.1%)	225 (21.3%)	87 (19.8%)	224 (25.5%)	237 (21.5%)	
<10/day	365 (9.7%)	36 (12.3%)	51 (4.8%)	44 (10.0%)	91 (10.4%)	143 (13.0%)	
10–20/day	280 (7.4%)	27 (9.2%)	27 (2.6%)	47 (10.7%)	69 (7.9%)	110 (10.0%)	
≥ 20/day	309 (8.2%)	71 (24.2%)	21 (2.0%)	29 (6.6%)	47 (5.4%)	141 (12.8%)	
Hypertension status							
Normal	1531 (40.6%)	97 (33.1%)	533 (50.5%)	199 (45.2%)	307 (35.0%)	395 (35.8%)	**< 0.001**
Elevated	614 (16.3%)	48 (16.4%)	153 (14.5%)	57 (13.0%)	163 (18.6%)	193 (17.5%)	
Hypertension stage 1	845 (22.4%)	59 (20.1%)	219 (20.7%)	98 (22.3%)	221 (25.2%)	248 (22.5%)	
Hypertension stage 2	634 (16.8%)	77 (26.3%)	117 (11.1%)	66 (15%)	153 (17.4%)	221 (20%)	
Hypertension crisis	32 (0.8%)	6 (2.0%)	1 (0.1%)	0 (0.0%)	6 (0.7%)	19 (1.7%)	
Missing	114 (3.0%)	6 (2.0%)	33 (3.1%)	20 (4.5%)	27 (3.1%)	28 (2.5%)	
Diabetes status							
Non‐diabetes	1986 (52.7%)	102 (34.8%)	702 (66.5%)	254 (57.7%)	407 (46.4%)	521 (47.2%)	**< 0.001**
Prediabetes	1139 (30.2%)	99 (33.8%)	244 (23.1%)	125 (28.4%)	310 (35.3%)	361 (32.7%)	
Diabetes	286 (7.6%)	37 (12.6%)	52 (4.9%)	22 (5.0%)	71 (8.1%)	104 (9.4%)	
Poorly controlled diabetes	200 (5.3%)	30 (10.2%)	22 (2.1%)	13 (3.0%)	58 (6.6%)	77 (7.0%)	
Missing	159 (4.2%)	25 (8.5%)	36 (3.4%)	26 (5.9%)	31 (3.5%)	41 (3.7%)	

*Note*: BMI, body mass index (calculated as weight [kg] divided by height squared [m^2^]). Income–poverty ratio is calculated by dividing total family income by the poverty threshold based on poverty guidelines, specific to family size, year and state. Hypertension status is categorised based on examined systolic and diastolic blood pressure values (SBP and DBP). Normal: SBP < 120 mmHg and DBP < 80 mmHg; Elevated: SBP 120–129 mmHg and DBP < 80 mmHg; hypertension stage I: SBP 130–139 mmHg or DBP 80–89 mmHg; hypertension stage II: SBP ≥ 140 mmHg or DBP ≥ 90 mmHg; hypertension crisis: SBP > 180 mmHg and/or DBP > 120 mmHg. Diabetes status is defined by tested glycohaemoglobin (HbA1c) levels. Non‐diabetes: HbA1c < 5.7%; Prediabetes: HbA1c 5.7%–6.4%; Diabetes: HbA1c ≥ 6.5%; Poorly controlled diabetes: HbA1c ≥ 8.0%. Bold values indicate statistical significance (*p* < 0.05).

Abbreviations: Caries + Periodontitis, individuals with both caries present and mild/moderate/severe periodontitis based on CDC/AAP definition; Caries, individuals with caries present and with no periodontitis based on CDC/AAP definition; Edentulous, individuals with no natural teeth present in oral cavity; Oral health, individuals with neither caries and nor periodontitis, using ‘no periodontitis’ based on CDC/AAP definition; Periodontitis, individuals with no caries and with mild/moderate/severe periodontitis based on CDC/AAP definition.

### 3.2 Oral‐Rinse Microbiota Profiles Across Oral Conditions

**FIGURE 1:**
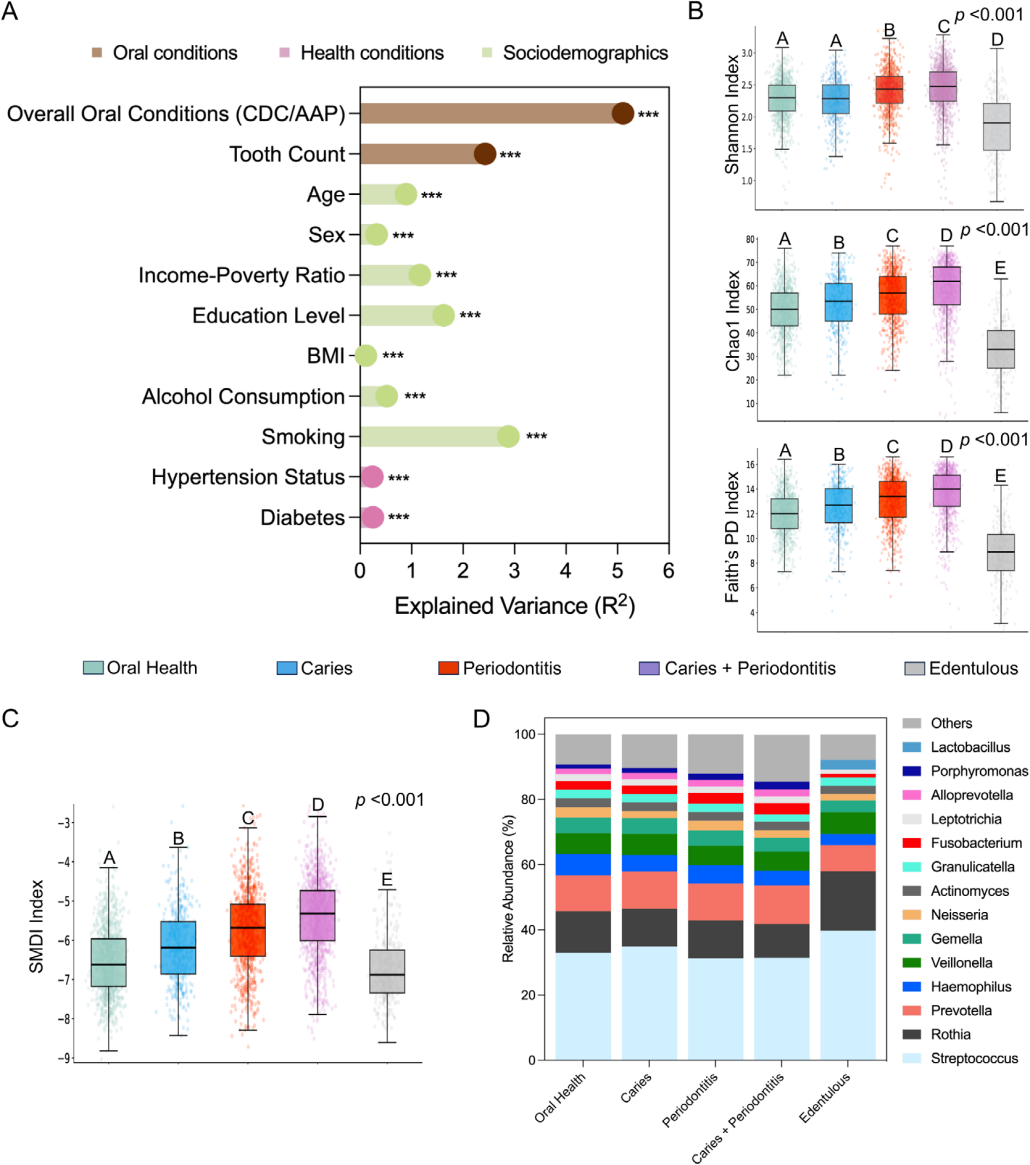
Association between oral conditions and oral microbiome. (A) Variance of microbial community based on the Aitchison dissimilarities from CLR transformed data, explained by oral conditions, sociodemographic factors and general health conditions. *R* ^2^ and *p*‐values were calculated by PERMANOVA test. (B) α‐Diversity comparisons across five oral conditions, measured by Shannon, Chao1 and Faith's PD indices. (C) Changes in periodontitis‐related microbiome dysbiosis across five oral conditions, measured by the subgingival microbial dysbiosis index (SMDI). (D) Average relative abundances of the most dominant taxa (> 1% average relative abundance) in different oral conditions. In (A), significance levels are denoted as follows: *p < 0.05, **p < 0.01, ***p < 0.001. For (B) and (C), the *p*‐value represents the results of the Kruskal–Wallis test across all groups. Post hoc pairwise comparisons were conducted using Dunn's test, with different letters above bars representing statistical differences (*p* < 0.05) and identical letters indicating no significant difference. Periodontitis status is defined based on the CDC/AAP definition. The comparison of α‐diversity and SMDI across different oral conditions accounted for the NHANES complex survey design.

Oral disease conditions were the strongest explanatory factor for microbial community variation, based on Aitchison distances (*R* ^2^ = 5.11%, *p* < 0.001; Figure 1A). Tooth count (*R* ^2^ = 2.43%, *p* < 0.001) and smoking status (*R* ^2^ = 2.88%, *p* < 0.001) also explained a substantial proportion of variance, whereas socio‐demographic and other‐health‐related factors accounted for less variation. The corresponding explained variance estimates based on Bray–Curtis and UniFrac dissimilarities are shown in Figure S2, and detailed variance partitioning across individual oral disease categories is provided in Figure S3.

α‐Diversity varied significantly across oral condition groups (Figure 1B). Edentulous individuals displayed the lowest α‐diversity, while the co‐existing diseases group had the highest diversity across all three indices. Groups with periodontitis (either alone or co‐existing with caries) showed significantly higher diversity than orally healthy, while caries‐only was intermediate.

SMDI further underscored these group‐level distinctions (Figure 1C); it was elevated in oral diseases, especially for periodontitis‐related groups (either alone or co‐existing with caries). This is consistent with the SMDI's development for periodontitis‐related subgingival dysbiosis. Meanwhile, orally healthy and edentulous individuals had significantly lower SMDI values.

Dominant taxa varied across oral conditions (Figure 1D). Edentulous individuals showed distinct shifts, with *Lactobacillus* emerging among the dominant taxa, *Porphyromonas* and *Alloprevotella* falling below 1% and increased proportions of *Streptococcus* and *Rothia*. Among dentate groups, the composition was relatively stable: *Streptococcus*, *Rothia* and *Prevotella* remained the most abundant genera (> 50% combined), while the diseased groups harboured a larger fraction of low‐abundance (< 1%) taxa. Results based on the ACES definition are presented in Figures S4 and S5.

### 3.3 Associations and Changes in Oral‐Rinse Microbiota Across Oral Conditions

**FIGURE 2:**
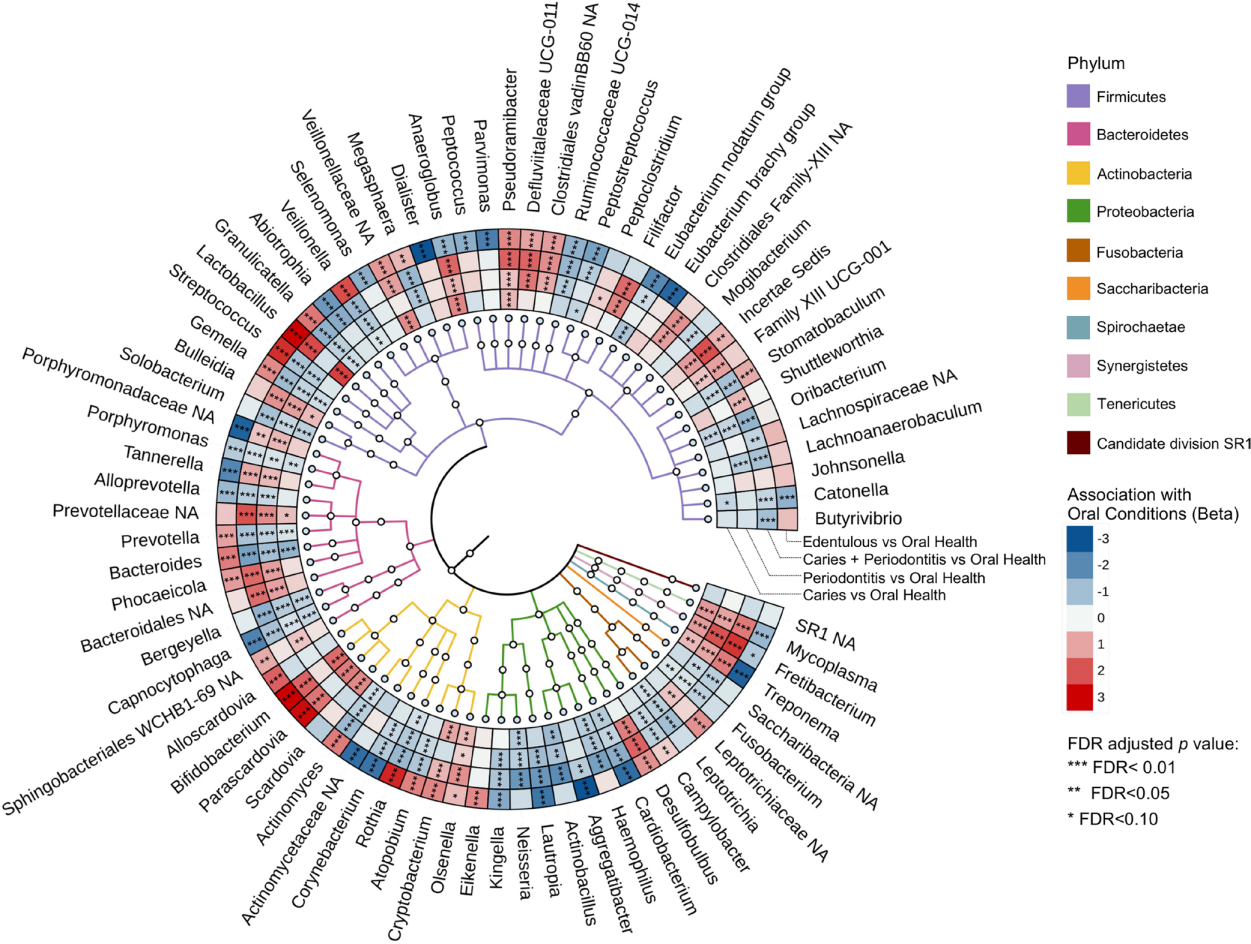
Association between oral rinse microbiota and oral conditions visualised by phylogenetic tree. The diagram illustrates the taxa positively associated (blue boxes in the outer wheels) and negatively associated (red boxes) in relation to oral health compared to edentulism, caries, periodontitis or the co‐occurrence of caries and periodontitis within microbial phylogenetic trees. In the heatmap, colour intensity indicates the strength and direction of associations between taxa and oral conditions (each compared to oral health). Asterisks within each box indicate the significance of associations: *p < 0.05, **p < 0.01, ***p < 0.001. The associations were evaluated by the MaAsLin2 approach, based on CLR‐transformed data, and adjusted for sex, race/ethnicity, body mass index, income–poverty ratio, education level, diabetes, and hypertension status. *p*‐values have been adjusted for multiple comparisons using the false discovery rate (FDR) method with a target rate of 0.1. Periodontitis status is defined based on the CDC/AAP definition. Colours on the phylogenetic tree's clade denote different bacterial phyla.

Multiple taxa were associated with oral disease conditions compared with oral health in the MaAsLin2 analyses (Figure 2). The two oral condition groups with periodontitis present were characterised by consistent positive associations of anaerobic taxa, including *Treponema*, *Filifactor*, *Tannerella* and *Fretibacterium*. Taxa commonly associated with oral health, such as *Streptococcus*, *Rothia* and *Actinomyces*, showed negative associations with disease groups. The caries‐only and co‐existing caries and periodontitis groups exhibited partial overlap (e.g., positive associations with *Lactobacillus*, *Parascardovia*, *Pseudoramibacter*), while other associations were group‐specific. Meanwhile, the edentulous group showed more divergent microbial associations. In Model 2, the direction of disease‐associated taxa remained largely consistent (Figure S6). However, the magnitude and statistical significance of associations were modestly reduced for caries‐ and periodontitis‐related disease groups. In contrast, associations with edentulism largely changed more substantially, likely because adjustment for tooth numbers directly influenced the comparison between edentulous and orally healthy groups. Results based on the ACES definition are presented in Supplementary Spreadsheet 3.

**FIGURE 3:**
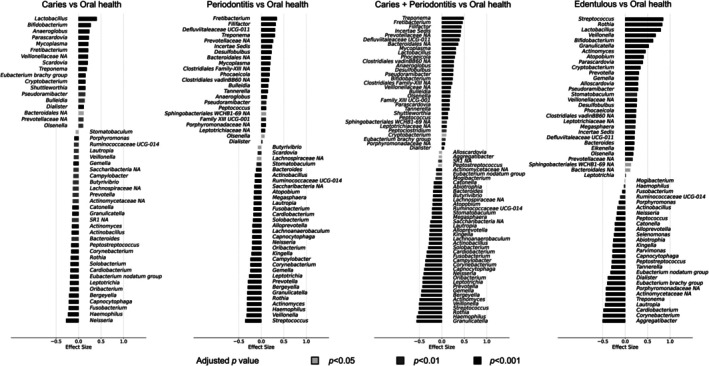
Differential abundance of taxa across oral healthy and diseased groups. Bar plots displaying differential abundance comparisons between each oral condition and oral health group by ALDEx2. Taxa with adjusted *p*‐value < 0.05 are shown in the plot. Analyses were performed with a centred log‐ratio (CLR) transformation of the count data with Monte Carlo sampling from the Dirichlet distribution. The estimated effect size was directly used for visualisation, representing the between‐group difference in CLR abundance. Analyses were adjusted by false discovery rate (FDR) for multiple testing. Oral conditions compared to oral health included caries‐only, periodontitis‐only, co‐existing caries and periodontitis and edentulous status. Periodontitis status is defined based on the CDC/AAP definition. Detailed ALDEx2 results are provided in Supplementary Spreadsheet 1.

Differential abundance analysis using ALDEx2 identified distinct microbial signatures across oral disease conditions (Figures 3 and S8). The caries‐only group was characterised by enrichment of acidogenic taxa, including *Lactobacillus*, *Bifidobacterium*, *Parascardovia* and *Scardovia*, accompanied by reductions in *Neisseria* and *Rothia*. In the periodontitis‐only group, anaerobic and inflammation‐associated taxa such as *Fretibacterium*, *Filifactor*, *Defluviitaleaceae* UCG‐011, *Treponema*, *Desulfobulbus*, *Mycoplasma*, *Tannerella* and *Pseudoramibacter* were consistently enriched, whereas the health‐associated genera *Rothia*, *Streptococcus*, *Actinomyces* and *Neisseria* were abundant in the healthy group. The co‐existing caries and periodontitis group showed combined enrichment of both caries‐ and periodontitis‐related taxa, including *Treponema*, *Filifactor*, *Defluviitaleaceae UCG‐011*, *Mycoplasma*, *Lactobacillus*, *Anaeroglobus*, *Desulfobulbus*, *Pseudoramibacter*, *Bifidobacterium*, *Olsenella*, *Parascardovia* and *Tannerella*, with reductions in *Rothia*, *Streptococcus*, *Actinomyces*, *Bergeyella* and *Neisseria*. In contrast, edentulous individuals exhibited a distinct abundance profile. Many taxa associated with caries or periodontitis showed significantly reduced abundance, while *Streptococcus*, *Rothia* and a subset of disease‐related genera (*Lactobacillus*, *Bifidobacterium*, *Parascardovia*, *Desulfobulbus*) were abundant, mirroring the dominant taxa patterns in Figure 1D.

Compared with ALDEx2 analyses, DESeq2 identified a larger number of disease‐abundant taxa and fewer health‐abundant taxa in comparisons involving caries‐ and periodontitis‐related groups versus oral health (Figures S7 and S9). Also, for edentulous comparisons, DESeq2 showed a higher number of health‐abundant taxa relative to ALDEx2. Complete results from ALDEx2 and DESeq2 analyses are provided in Supplementary Spreadsheets 1 and 2.

### 3.4 Severity‐Dependent Changes in Oral‐Rinse Microbiota for Periodontitis

Dentate participants without caries were further stratified by periodontitis severity. Socio‐demographic and systemic health factors shifted in parallel with increasing periodontitis severity under both definitions. Detailed population characteristics are provided in Tables S2 and S3.

**FIGURE 4:**
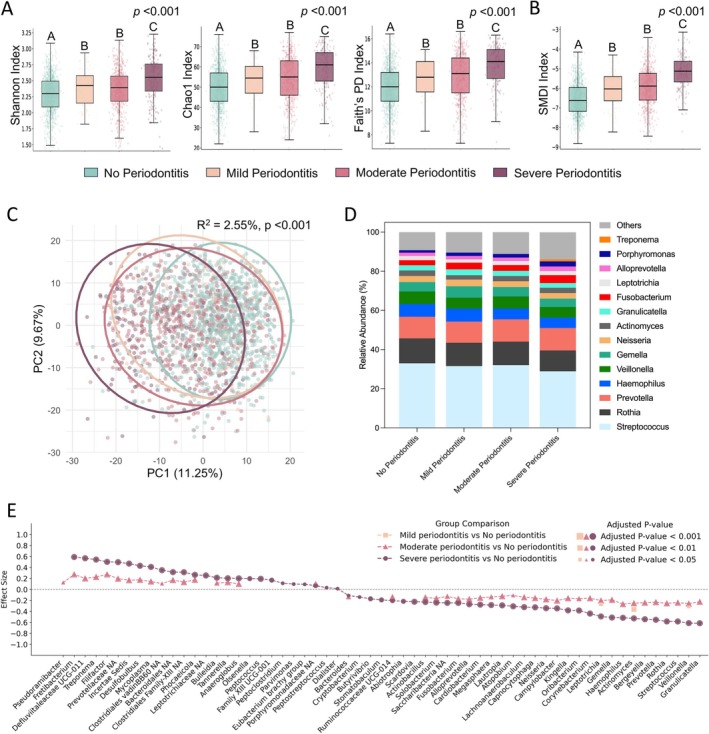
Dose‐dependent association between oral microbiome profiles and periodontitis severity. (A) α‐Diversity comparisons across periodontitis severity status, measured by Shannon, Chao1 and Faith's PD indices. (B) Changes in periodontitis‐related microbiome dysbiosis across periodontitis severity status, measured by the subgingival microbial dysbiosis index (SMDI). (C) Principal component analysis (PCA) based on Aitchison dissimilarities of CLR‐transformed data, comparing microbial community structures across periodontitis severity status. Each point represents an individual; ellipses indicate 95% confidence regions. Explained variance (*R* ^2^) and *p‐*values were calculated by PERMANOVA test. (D) Average relative abundances of the most dominant taxa (> 1% average relative abundance) across periodontitis severity status. (E) Differential abundance comparisons between relatively healthy periodontal status (no periodontitis in CDC/AAP definition) and more advanced periodontitis stages. Each dot represents a taxon with significant changes, with dot position indicating the magnitude of change (effect size estimated from between‐group CLR differences) and dot size reflecting adjusted *p*‐value significance. Statistical significance was assessed using Welch's *t*‐test and Wilcoxon rank test on Monte Carlo samples generated by ALDEx2 on CLR‐transformed data, followed by false discovery rate (FDR) correction for multiple testing. Only taxa with adjusted *p*‐values < 0.05 are shown in the plot. For (A) and (B), *p*‐values represent Kruskal–Wallis test results across all groups. Post hoc pairwise comparisons were conducted using Dunn's test; different letters above bars indicate statistically significant differences (*p* < 0.05), while identical letters indicate no significant difference. Periodontitis status is defined based on the CDC/AAP definition. The comparison of α‐diversity and SMDI across different oral conditions accounted for the NHANES complex survey design.

α‐Diversity increased progressively from no periodontitis to severe across all three indices (Figure 4A), and SMDI showed the same trend (Figure 4B), but differences between mild and moderate were not significant. β‐Diversity analyses supported these patterns: microbial community variance across all severities was significant (*R* ^2^ = 2.55%, *p* < 0.001; Figure 4C), with larger variances seen between severe groups and the other groups, whereas mild versus moderate showed no significant variance (Figure S10A). Dominant taxa were stable across severities (Figure 4D), with a notable shift in the severe periodontitis group where *Treponema* emerged among the dominant taxa. And the proportion of low‐abundance (< 1%) taxa increased with severity.

Differential abundance assessed by ALDEx2 identified taxa with dose‐dependent changes across periodontitis severity (Figure 4E). Fifty‐nine taxa showed significant changes from no periodontitis through mild, moderate and severe periodontitis. Many of these are well‐recognised taxa of subgingival dysbiosis in periodontitis, including *Fretibacterium*, *Treponema*, *Filifactor*, *Desulfobulbus*, *Tannerella*, *Anaeroglobus*, *Peptococcus* and *Parvimonas*. Additional results based on ACES definitions as well as results from non‐compositional analyses are provided in Figures S10–S12 and Supplementary Spreadsheets 4 and 5.

### 3.5 Diagnostic Potential of Oral‐Rinse Taxa for Periodontitis

**FIGURE 5:**
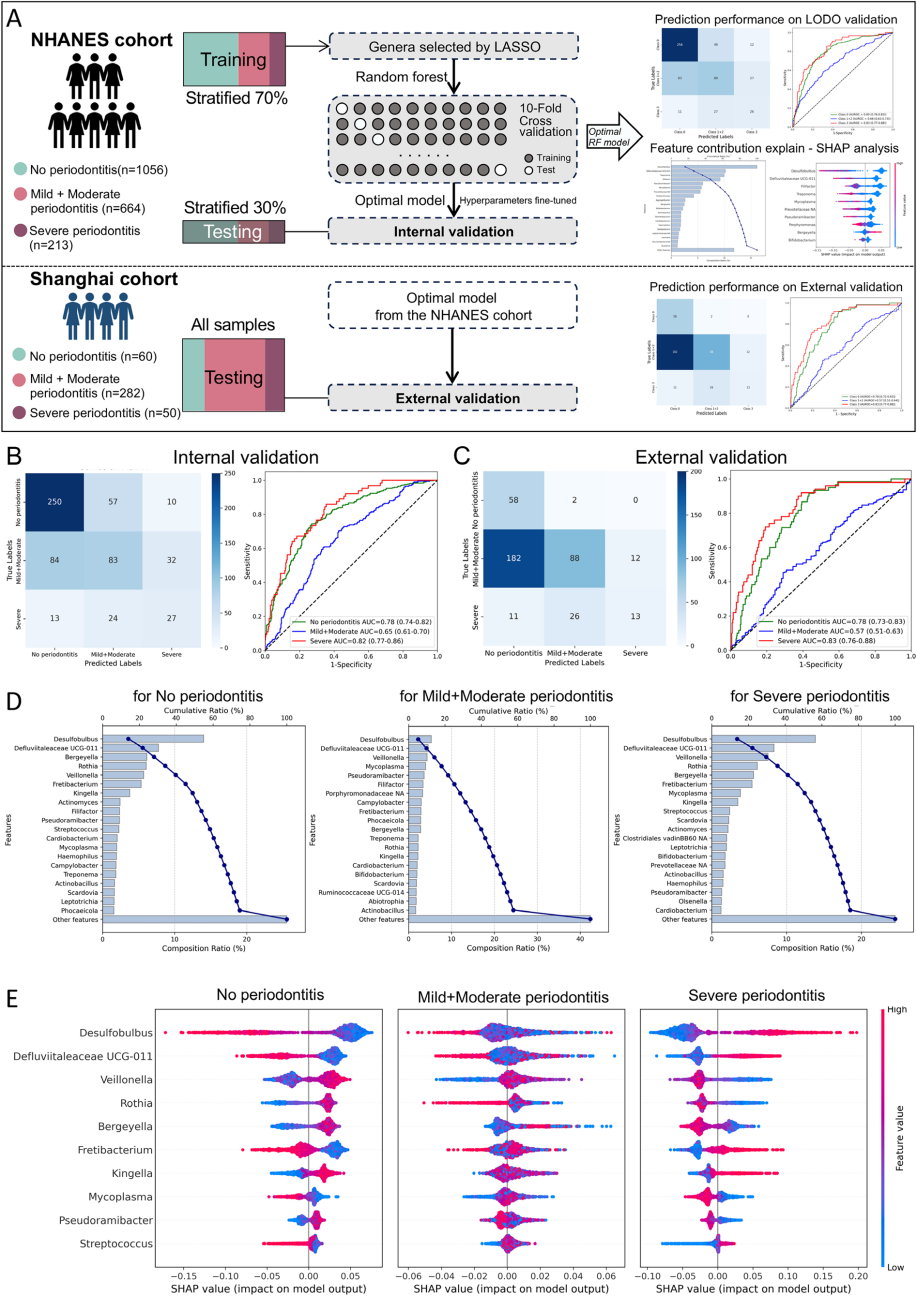
Machine learning model for periodontitis prediction based on microbial taxa. (A) Framework for data partitioning, model training, independent held‐out test set for internal validation and external validation, utilising genus‐level features selected by LASSO regression for model construction. Periodontitis status is defined based on the CDC/AAP definition, and CLR‐transformed data was used for model development and validation. (B) Model performance of the optimal multi‐class model for classifying periodontitis severity in internal validation with independent held‐out test set, including confusion matrix and receiver operating characteristic (ROC) curves. Confusion matrix displays the performance of the trained random forest multi‐class model in internal validation with independent held‐out test set. (C) Model performance of the optimal multi‐class model for classifying periodontitis severity in external validation with the investigator‐initiated cohort (Shanghai cohort). (D) Pareto plots showing the top‐contributing taxa with single and cumulative contribution to model interpretability, as determined by the random forest multi‐class model and Shapley additive explanation (SHAP). (E) SHAP summary plots for the top 10 taxa contributing to model interpretability. Each point represents a sample, coloured by the CLR‐transformed relative abundance of the taxon (blue to red representing low to high abundance). The *x*‐axis shows the SHAP value, indicating the magnitude and direction of each taxon's impact on the model output.

Machine learning was used to develop diagnostic models for classifying periodontitis from oral‐rinse taxa. LASSO was applied for feature selection, yielding 60 taxa for the CLR‐transformed data‐based model and 34 taxa for the relative abundance data‐based model, which were then used to develop the multi‐class RF models (Figure 5A).

In internal validation for the test set, the optimal CLR‐based model showed acceptable performance for severity classification (Figure 5B). AUROC was 0.78 (95% CI: 0.74–0.82) for no periodontitis and 0.81 (0.75–0.87) for severe periodontitis, while performance for the combined mild + moderate group was lower at 0.65 (0.60–0.69). Models trained on relative abundance data showed similar internal performance (Figure S13B).

In external validation with the independent cohort, the same optimal CLR‐based model performed similarly or slightly better (Figure 5C). AUROC was 0.78 (0.72–0.83) for no periodontitis and 0.83 (0.77–0.88) in the severe group, while performance in the intermediate group declined modestly to 0.57 (0.51–0.64). Model trained on the CLR‐transformed data showed relatively consistent performance between internal and external validations, while the model trained on relative abundance data exhibited reduced performance in external validation (Figure S13). Population characteristics of 392 participants in the local cohort are provided in Table S4.

Model interpretation with SHAP showed that, across CLR‐based and relative‐abundance‐based models, the top 10 taxa accounted for ~60%–70% of the total feature importance in the least and most severe categories and ~40%–50% in the intermediate category (Figures 5D and S14). Leading features in the CLR‐based model included *Desulfobulbus*, *Defluviitaleaceae* UCG‐011, *Veillonella*, *Rothia*, *Bergeyella*, *Fretibacterium*, *Kingella*, *Mycoplasma*, *Pseudoramibacter* and *Streptococcus*, including many periodontal health‐associated taxa, consistent with earlier differential abundance patterns (Figure 5E). By contrast, the relative‐abundance‐based model emphasised a narrower set of taxa predominantly associated with periodontitis, particularly in the least and most severe categories. ACES‐definitions‐related results are provided in Figures S13 and S15.

## 4 Discussion

This study provides a comprehensive analysis of oral‐rinse‐derived microbiota across oral conditions considering periodontitis, dental caries, and edentulism, within a representative US population. Oral‐rinse samples integrate microbes from diverse oral niches, offering a composite view of microbiota dynamics. Our findings indicate that oral diseases explain more variance in the oral‐rinse microbial community than other host factors, underscoring the value of examining oral‐condition‐specific microbial shifts. To our knowledge, this is the first large‐scale study to comprehensively characterise oral‐rinse microbiota profiles across distinct oral conditions accounting for both caries and periodontitis and to evaluate the potential of oral‐rinse microbiota for distinguishing periodontitis severity.

As reported, two classification systems were applied for periodontitis. Both CDC/AAP and ACES captured disease‐associated microbial shifts. However, ACES exhibited imbalanced group sizes especially for periodontal health and early disease because of its inclusive criteria for periodontitis (Holtfreter et al. 2024), which is consistent with prior observations (Tay et al. 2025).

In edentulism, microbial shifts were expected: tooth loss removes these hard surfaces for the biofilm, restructuring microbial habitat toward mucosa, dentures and the tongue dorsum. This was accompanied by reduced α‐diversity and a distinct microbial community, aligning with previous saliva findings (Gazdeck et al. 2019). Most taxa decreased, while, notably, caries‐associated aciduric genera persisted, likely because of their ability to adhere to mucosal/denture surfaces and tolerance of acidic environments, highlighting microbial adaptability to anatomical changes.

For caries, localised supragingival niches typically accumulate acidogenic/aciduric bacteria, shifting plaque towards pathogenic biofilms and often lowering diversity under acid stress (Lamont et al. 2018; Peterson et al. 2011). In our results, the caries‐only group showed similar or even higher α‐diversity than the oral health group. This may reflect the composite nature of oral rinse, which samples diseased and healthy sites simultaneously, while caries‐associated taxa may modestly increase the overall diversity. Differential abundance results showed abundant caries‐associated taxa (*Lactobacillus*, *Bifidobacterium*, *Pseudoramibacter*, *Dialister* and *Scardovia*) and declines in health‐associated taxa (*Rothia*, *Neisseria*, *Haemophilus* and *Capnocytophaga*) (Baker et al. 2021; Lamont et al. 2018). By contrast, saliva‐based studies have reported fewer differentially abundant taxa between caries‐active and healthy individuals (Yang et al. 2012), suggesting that oral rinse may better capture broader microbial signals; however, confirmation in matched cohorts is needed.

Oral‐rinse microbiota in periodontitis showed patterns resembling subgingival plaque, both in periodontitis alone and co‐existing with caries. α‐Diversity and SMDI were significantly elevated across oral condition groups and increased with periodontitis severity. Differential abundance results also overlapped with previously reported taxa enriched in periodontitis (Feres et al. 2021; Meuric et al. 2017), while periodontal‐health‐associated genera were reduced, in line with established cognitions. These findings support oral rinse as a potential tool for capturing subgingival shifts, aligning with recent work that did not account for caries status (Balan et al. 2025). Prior studies have shown that salivary microbiota partially reflect subgingival changes via gingival crevicular fluid, which helps in explaining why oral rinse can detect these signals (Belstrøm 2020; Lundmark et al. 2019).

In co‐existing caries and periodontitis, microbial profiles indicated complex dysbiosis. β‐Diversity showed community divergence. A distinct pattern emerged with concurrent enrichment of periodontitis‐associated taxa (*Filifactor*, *Treponema*, *Fretibacterium*, *Tannerella* and *Porphyromonas*) and caries‐associated genera (*Lactobacillus*, *Bifidobacterium*, *Parascardovia* and *Scardovia*), alongside consistent reduction in health‐associated taxa (*Haemophilus*, *Rothia*, *Neisseria* and *Bergeyella*). Interestingly, community dissimilarity indicated that the co‐existing group more closely resembled periodontitis than caries. Together, these results suggest that oral rinse captures overlapping microbial signatures of dual pathologies and may more effectively reflect periodontitis status, even though key periodontitis‐associated taxa primarily reside in the subgingival plaque.

Building on the ability of oral rinse to reflect periodontitis‐related shifts, we evaluated its potential utility for disease classification. Using genus‐level data, the machine learning model achieved moderate diagnostic performance for severe periodontitis, while performance for intermediate disease stages was more limited. Although the model performance was lower than those reported for SMDI (Chen et al. 2022), it should be noted that SMDI was originally developed to distinguish moderate to severe periodontitis from no periodontitis while excluding mild periodontitis, limiting its applicability across the full disease spectrum encountered in real‐world populations. Notably, the model trained on CLR‐transformed data demonstrated better generalisability in external validation. This mirrors previous findings in gut‐microbiome‐based disease prediction (Li et al. 2023), supporting the cross‐cohort robustness of compositional approaches. Model interpretability analyses further supported biological plausibility, highlighting well‐known periodontitis‐associated genera and the health‐associated genus (Feres et al. 2021; Meuric et al. 2017).

Performance for intermediate disease stages was poor to fair, reflecting the transitional nature of mild to moderate periodontitis, which lacks a clearly separable microbial signature. This was supported by SHAP analyses, where genera such as *Fretibacterium*, *Mycoplasma*, *Pseudoramibacter* and *Kingella* showed inconsistent or bidirectional contributions, underscoring the difficulty in reliably classifying intermediate states. The co‐existence of health‐ and disease‐associated species within the same genera further limits discriminative power at this taxonomic resolution (Chen et al. 2018; López‐López et al. 2017). Several limitations of this study, however, should be acknowledged. The NHANES dataset provided only genus‐level information, restricting insights into species‐level associations and constraining the diagnostic power of machine learning models. In addition, limited clinical information on caries severity, bleeding on probing and denture use reduced the disease granularity and limited sensitivity analyses in specific subsets. The age restrictions on oral microbiome sequencing and periodontal examination in NHANES limited generalisability to younger and older populations. Importantly, NHANES provided only taxonomically assigned read count tables, without access to raw sequencing reads, ASV‐level data or batch information, precluding reprocessing with updated reference databases or batch correction and potentially affecting cross‐cohort comparability and external validation performance.

Taken together, these findings indicate that oral‐rinse microbiota can capture meaningful disease‐related signals, particularly for advanced periodontitis. This characteristic may be valuable for large‐scale or population‐based studies that require non‐invasive, easily collected biospecimens. The moderate performance observed for severe disease and the limited discrimination of intermediate stages from our machine learning model suggest that oral‐rinse microbiota alone is not yet sufficient for reliable clinical severity classification. Future studies integrating oral‐rinse microbiota with established clinical indicators, higher resolution taxonomic data and more advanced modelling approaches will be necessary before considering clinical implementation.

## Author Contributions

M.S.T., B.T.R. and Y.X. conceived and designed the study. Y.X. and A.A. analysed the data with X.Y., M.B., H.L. and Y.L. Y.X., B.T.R. and M.S.T. drafted the manuscript with input from A.R. and A.M. All authors have reviewed the final version of the manuscript and approved it for publication.

## Conflicts of Interest

The authors declare no conflicts of interest.

## Supporting information


**Data S1:** jcpe70101‐sup‐0001‐Supinfo.docx.

**Data S2:** Supplementary Spreadsheet 1. Differential abundance by ALDEx2 in different oral conditions.

**Data S3:** Supplementary Spreadsheet 2. Differential abundance by DESeq2 in different oral conditions.

**Data S4:** Supplementary Spreadsheet 3. MaAsLin2 results for linear associations between CLR‐transformed genus‐level data and oral conditions defined by caries status and the ACES periodontitis classification, under multiple covariate‐adjusted models.

**Data S5:** Supplementary Spreadsheet 4. Taxa identified in the Venn comparison of ALDEx2 and DESeq2 differential abundance analyses under identical oral condition contrasts.

**Data S6:** Supplementary Spreadsheet 5. Taxa identified in the Venn comparison of ALDEx2 and DESeq2 differential abundance analyses under identical periodontitis status contrasts.

## Data Availability

The data that support the findings of this study are available on request from the corresponding author. The data are not publicly available due to privacy or ethical restrictions.
