# Supplementary material for: Oral‐Rinse‐Sourced Microbiota in Oral Health and Diseases in a Representative US Adult Population: Implications for Diagnostics
Source: J Clin Periodontol. 2026 Jan 14;53(5):760–73. doi: 10.1111/jcpe.70101 (PMC13086544; doi:10.1111/jcpe.70101)
Supplement: Supplementary file 1 — Data S1: jcpe70101‐sup‐0001‐Supinfo.docx. [file JCPE-53-760-s004.docx]

**Supplementary Methods**

***Oral Health Status Definitions***

For the NHANES database, oral health examinations were conducted by dental examiners who were dentists licensed in at least one U.S. state. A health technician entered examiner observations directly into a computerized data-collection system. All assessments took place in a dedicated room within the Mobile Examination Center (MEC) equipped with a portable dental chair, dental light, and compressed air. Clinical protocols followed standardized NHANES procedures(Centers for Disease Control and Prevention & Statistics, 2024a, 2024b).

All participants first underwent a tooth-count assessment, followed by evaluation for dental caries. For the purposes of this study and with the limitation of available NHANES data, a participant was classified as having caries if the NHANES data indicated at least one tooth with dental decay. Notably, caries assessment protocols differed across the two survey cycles analyzed in this study: The 2009-2010 cycle provided only a binary indicator of the presence of at least one decayed tooth; detailed examination procedures were not documented in the public manual for that cycle. The 2011-2012 cycle provided the NHANES Oral Health Examiners Manual, describing a standardized caries assessment for all teeth except third molars. Quadrants were dried with air as needed. Examiners used a surface-reflecting mirror and a No. 23 explorer. Frank lesions were identified as gross cavitation. Surfaces assessed were lingual, facial (buccal), mesial, and distal for anterior teeth, and lingual, occlusal, facial (buccal), mesial, and distal for posterior teeth. For incipient proximal lesions, a positive diagnosis required detecting a break in the enamel surface with the explorer in posterior teeth, while a positive diagnosis required either explorer-detected enamel break or clear visualization by transillumination in anterior teeth.

Periodontal examination protocols were consistent across both cycles. Probing pocket depth and gingival recession were measured with a periodontal probe by the dental examiners; clinical attachment loss was calculated as pocket depth minus gingival recession. Examinations were full-mouth (excluding third molars), with six sites per tooth assessed: distofacial, midfacial, mesiofacial, distolingual, midlingual, and mesiolingual.

Periodontitis status was defined separately using two established systems: the Centers for Disease Control and Prevention–American Academy of Periodontology (CDC/AAP) classification system and the Application of the 2018 Periodontal Status Classification to Epidemiological Survey data (ACES) system.

The definitions of different levels’ periodontitis under CDC/AAP classification system are as follows(Eke et al., 2012):

(i) no periodontitis: individual absence of mild, moderate, or severe periodontitis found;

(ii) mild periodontitis: an individual with ≥2 interproximal sites with attachment loss ≥3 mm, and ≥2 interproximal sites with pocket depth ≥4 mm (not on the same tooth) or one site with pocket depth ≥5 mm;

(iii) moderate periodontitis: an individual with ≥2 interproximal sites showing attachment loss of ≥4 mm (not on the same tooth) or ≥2 interproximal sites exhibiting pocket depth of ≥5 mm (not on the same tooth);

(iv) severe periodontitis: an individual with ≥2 interproximal sites with attachment loss ≥6 mm (not on the same tooth) and ≥1 interproximal site with pocket depth ≥5 mm.

The definition of different levels’ periodontitis under ACES classification system are as follows(Holtfreter et al., 2024):

(i) no periodontitis: an individual with no interproximal attachment loss ≥1 mm at ≥2 non-adjacent teeth and no buccal/lingual attachment loss ≥3 mm with pocket depth >3 mm at ≥2 teeth is observed;

(ii) stage I periodontitis: an individual with interproximal attachment loss ≥1 mm at ≥2 non-adjacent teeth, with maximum attachment loss of 1-2 mm;

(iii) stage II periodontitis: an individual with interproximal attachment loss ≥1 mm at ≥2 non-adjacent teeth, with maximum attachment loss of 3-4 mm;

(iv) stage III/IV periodontitis: an individual with interproximal attachment loss ≥1 mm at ≥2 non-adjacent teeth, with maximum attachment loss ≥5 mm.

Notably, under the ACES classification system, no participants met the criteria for "no periodontitis" in the 3477 individuals included in this study.

Following the definitions above, dentate individuals were grouped by the presence/absence of caries and periodontitis, as described in the main text: (i) oral health (neither caries nor periodontitis present), (ii) caries only (only caries present, without periodontitis), (iii) periodontitis only (only periodontitis present, without caries), and (iv) co-existing caries and periodontitis (both caries and periodontitis present). Because no participants qualified as “no periodontitis” under ACES, ACES stage I was used as the operational proxy for periodontal health when assigning “oral health” and “caries only” categories under ACES.

For the local cohort used for external validation, oral health examinations were conducted by calibrated examiners who were dentists licensed in China. After collecting the oral rinse samples, all participants first underwent a tooth-count assessment, followed by measurement of probing pocked depth, gingival recession and bleeding on probing using a standard periodontal probe. Clinical attachment loss was calculated as probing pocket depth minus gingival recession. Full-mouth periodontal examinations were performed excluding third molars, with six sites per tooth assessed: distofacial, midfacial, mesiofacial, distolingual, midlingual, and mesiolingual. Periodontitis status was defined separately using either the CDC/AAP or the ACES classification system, consistent with the definitions applied in the NHANES database.

***Oral Rinse Collection and 16S rRNA Gene Sequencing***

In NHANES, oral rinse samples were collected at the MECs before the oral health examination. Trained dental examiners instructed participants to rinse and gargle with 10 mL of saline mouthwash for 30 seconds. Sample DNA extraction and 16S rRNA gene amplicon sequencing were performed in certified laboratories as previously described (Centers for Disease Control and Prevention & Statistics). Detailed sequencing and data processing procedures and results followed the NHANES Oral Microbiome Data Documentation (<https://wwwn.cdc.gov/nchs/data/nhanes/omp/OralMicrobiomeDataDocumentation-508.pdf>).

Briefly, The V4 region of the 16S rRNA gene was amplified using primers 515F (5’-GTG CCA GCM GCC GCG GTA A-3’) and 806R (5’-GGA CTA CHV GGG TWT CTA AT-3’), producing ~390 bp amplicons. Sequencing was conducted on the Illumina HiSeq 2500 System using 2x125 bp paired-end reads, following the manufacturer’s protocols.

As described in the corresponding documentation, raw sequencing reads were demultiplexed using QIIME1 (version 1.9.1) to generate separate forward and reverse FASTQ files for each individual. Due to insufficient overlap between the 125 bp paired-end reads, only forward reads were retained for further analysis. Sequence processing was performed using the DADA2 pipeline (version 1.2.1), which included quality filtering, error correction, chimera removal, and inference of amplicon sequence variants (ASVs). Each sequencing run was processed independently using DADA2 and ASV tables, and the results were subsequently merged. The taxonomic assignment of ASVs was carried out using the SILVA v123 database. Relative abundance and read count tables were generated with QIIME 1.9.1 without rarefying the data.

In the local cohort, oral rinse samples were collected by calibrated examiners before periodontal examination. Participants were instructed to rinse and gargle with 5 mL of distilled water for 30 seconds. DNA was extracted from the samples, and 16S rRNA gene amplicon sequencing was performed following standard protocols. The V3-V4 hypervariable region of the 16S rRNA gene was amplified using primers 341F (5’-CCT AYG GGR BGC ASC AG-3’) and 806R (5’-GGA CTA CNN GGG TAT CTA AT-3’), generating ~460 bp amplicons. Sequencing was conducted on the Illumina NovaSeq 6000 platform with 2x250 bp paired-end reads, according to the manufacturer’s instructions. Raw sequencing reads were processed using QIIME2 (version 2025.4.0), including demultiplexed, quality filtering, and denoising with the DADA2 pipeline to generate ASVs. Taxonomic assignment of ASVs was performed with SILVA v138.1 database, using the pretrained official naive Bayesian classifier. Feature tables were collapsed to genus level, and read count tables were generated without rarefaction to mirror the NHANES processing workflow. These tables were then used as the external-validation dataset.

***Statistical Analyses***

For descriptive statistics analyses of population characteristics and clinical parameters across groups, the normality of continuous variables was assessed with the Shapiro–Wilk test. As distributions were non-normal, continuous variables are presented as median (interquartile range, IQR) and categorical variables as counts (n) and percentages (%). Group differences used the Kruskal–Wallis test for continuous variables (with Dunn’s post hoc pairwise comparisons) and chi-square tests for categorical variables, accounting for the complex NHANES survey design with the Survey package in R.

For microbiome analyses, taxa were excluded if they were not present with >10 reads in ≥30% of individuals in at least one of the oral conditions (oral health, caries, periodontitis, caries & periodontitis, or edentulism), or lacking taxonomic annotation at the Class level. In addition, samples with <5000 reads were removed. All microbiome analyses and visualizations were conducted using the filtered dataset (**Supplemental Fig. 1**).

Alpha diversity indices (Shannon and Chao1) were calculated using the Vegan package in R (J. Oksanen et al., 2017), whereas Faith’s phylogenetic diversity (PD) was calculated using the Picante package based on the corresponding phylogenetic tree. The subgingival microbial dysbiosis index (SMDI) was calculated using the online SMDI platform(Chen et al., 2022)^[[1]](#footnote-1)^&. All alpha diversity metrics and SMDI were calculated using read count tables. Intergroup comparisons were performed using Kruskal-Wallis test, followed by pairwise post-hoc analysis using Dunn’s test with Bonferroni correction, accounting for the complex NHANES survey design with the Survey package in R.

For beta diversity analysis, both compositional and non-compositional approaches were applied, with non-compositional analyses as sensitivity analyses. Compositional analyses were conducted using CLR-transformed data, with Aitchison distances calculated to assess community dissimilarity. Statistical differences were evaluated using permutational multivariate analysis of variance (PERMANOVA), and the coefficient of determination (R²) was reported as the proportion of variance explained. The Aitchison distances were visualized using principal component analysis (PCA). Non-compositional analyses were performed as sensitivity analyses using read count data, with Bray-Curtis dissimilarities, weighted UniFrac distances and unweighted UniFrac distances calculated. Explained variance were assessed using PERMANOVA, and the results were visualized using principal coordinates analysis (PCoA).

Associations between microbial taxa and oral health conditions were evaluated using Microbiome Multivariate Association with Linear Models (MaAsLin2), with the orally healthy group serving as the reference. CLR-transformed data were used for all MaAsLin2 analyses. Two covariate-adjusted models were applied: Model 1 adjusted for sex, race/ethnicity, body mass index (BMI), income-to-poverty ratio, education level, hypertension status, and diabetes status; Model 2 additionally adjusted for age, smoking status and tooth numbers. *P* values were adjusted using the Benjamini-Hochberg false discovery rate (FDR), with a significance threshold set at FDR < 0.1. Associations were visualized in combination with phylogenetic information using the Interactive Tree of Life (iTOL, version 7.1)

For differential abundance analyses, compositional method was applied using the ALDEx2 package in R as the main analyses. Comparisons were performed between the oral health group and each diseased group, with multiple testing correction conducted using the Benjamini–Hochberg procedure. As a sensitivity analyses, differential abundance analysis was additionally performed using DESeq2 on read count data. Differences in significant taxa identified by ALDEx2 and DESeq2 were compared using Venn diagrams.

For the machine learning pipeline, the NHANES dataset was stratified by periodontitis severity and randomly split into training (70%) and held-out testing (30%) subsets. To mitigate class imbalance under the CDC/AAP definition, mild and moderate periodontitis were combined into a single class of “mild and moderate periodontitis”. The machine learning analyses were performed separately using CLR-transformed data and relative abundance data. Feature selection was performed on the training set using least absolute shrinkage and selection operator (LASSO) as a dimensionality-reduction step. Prior to LASSO, features were standardized using z-score scaling. The LASSO regularization parameter was determined by internal cross-validation on the training data, and taxa with non-zero coefficients were retained for downstream modelling.

A random forest (RF) classifier was then developed using the selected taxa to classify periodontitis severity. No additional scaling or normalization was applied to the CLR-transformed or relative abundance features used for random forest training and evaluation. Class imbalance was addressed using class-weighted learning. RF hyperparameters were optimized using GridSearchCV with 10-fold cross-validation on the training set, targeting the optimal weighted F1 score. The optimal RF model was saved and subsequently applied to the held-out testing set and the independent local cohort for internal and external validation, respectively.

For external validation, the saved optimal RF model was applied to the local cohort without refitting. Taxa in the external dataset were aligned to the model’s feature set using a predefined mapping table. Taxa present in the trained model but not detected in the local cohort were treated as absent and assigned zero values prior to prediction.

Model performance was summarized using class-specific AUROC, accuracy, sensitivity and specificity, with 95% confidence intervals (CIs) derived via non-parametric bootstrap resampling. Specifically, 1,000 bootstrap replicates were generated by resampling the testing set with replacement, and performance metrics were recalculated for each replicate. Point estimates were obtained from the original evaluation datasets, and 95% CIs were derived from the bootstrap distributions. Bootstrap resampling treated the held-out test set as a simple random sample and did not incorporate the complex NHANES survey design. Model interpretation was assessed using Shapley Additive exPlanations (SHAP) analysis(Lundberg & Lee, 2017) , and results were visualized using Pareto rankings of feature contributions and SHAP summary plots.

Although NHANES recommends accounting for the complex survey design for population-representative inference, many commonly used microbiome analytical frameworks are not designed to incorporate survey weights. Accordingly, microbiome analyses in this study were performed at the sample level without applying NHANES survey weights, to ensure internal methodological consistency across diversity analyses, differential abundance and association analyses, and machine-learning modeling.

**Reference**

Centers for Disease Control and Prevention, & Statistics, N. C. f. H. *NHANES 2011-2012 Laboratory Data Overview*. Retrieved 2024 from <https://wwwn.cdc.gov/nchs/nhanes/continuousnhanes/overviewlab.aspx?BeginYear=2011>

Centers for Disease Control and Prevention, & Statistics, N. C. f. H. (2024a). *National Health and Nutrition Examination Survey (NHANES) 2009-2010*. Retrieved Accessed January 2024 from <https://wwwn.cdc.gov/nchs/nhanes/continuousnhanes/default.aspx?BeginYear=2009>

Centers for Disease Control and Prevention, & Statistics, N. C. f. H. (2024b). *National Health and Nutrition Examination Survey (NHANES) 2011-2012*. Retrieved Accessed January 2024 from <https://wwwn.cdc.gov/nchs/nhanes/continuousnhanes/default.aspx?BeginYear=2011>

Chen, T., Marsh, P. D., & Al-Hebshi, N. N. (2022). SMDI: An Index for Measuring Subgingival Microbial Dysbiosis. *J Dent Res*, *101*(3), 331-338. <https://doi.org/10.1177/00220345211035775>

Eke, P. I., Page, R. C., Wei, L., Thornton-Evans, G., & Genco, R. J. (2012). Update of the case definitions for population-based surveillance of periodontitis. *J Periodontol*, *83*(12), 1449-1454. <https://doi.org/10.1902/jop.2012.110664>

Holtfreter, B., Kuhr, K., Borof, K., Tonetti, M. S., Sanz, M., Kornman, K., Jepsen, S., Aarabi, G., Völzke, H., Kocher, T., Krois, J., & Papapanou, P. N. (2024). ACES: A new framework for the application of the 2018 periodontal status classification scheme to epidemiological survey data. *J Clin Periodontol*, *51*(5), 512-521. <https://doi.org/10.1111/jcpe.13965>

J. Oksanen, R. Kindt, P. Legendre, B. O'Hara, G. Simpson, P. Solymos, M. Stevens, & Wagner., H. (2017). *vegan: Community Ecology Package*. In (Version 2.4-2) <http://CRAN.R-project.org/package=vegan>

Lundberg, S. M., & Lee, S.-I. (2017). A unified approach to interpreting model predictions. *Advances in neural information processing systems*, *30*, 4765-4774.

**Supplementary Figures**


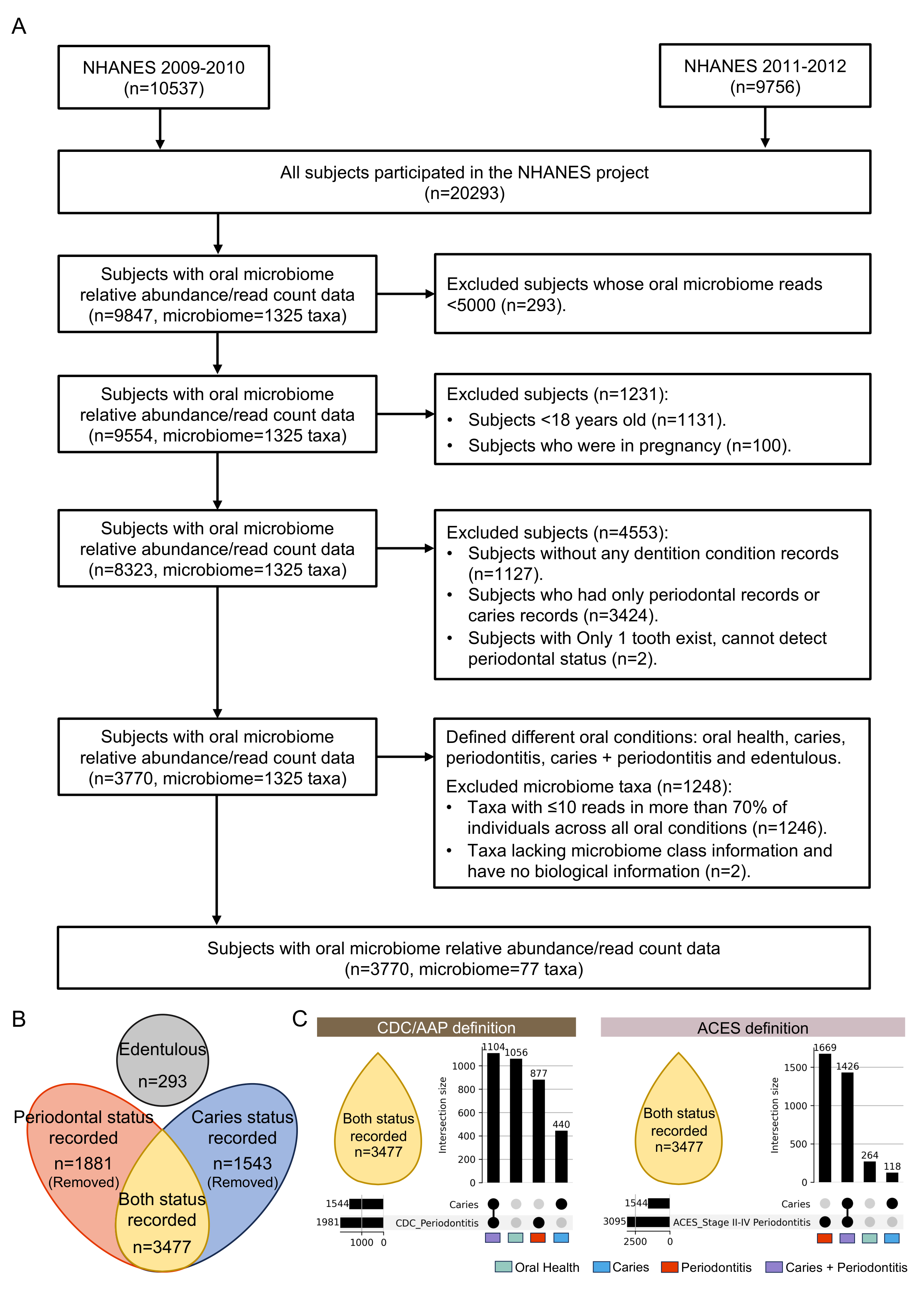


**Figure S1 Flow chart of subject and microbiome inclusion process from the NHANES 2009-2010 and 2011-2012 database**

(A) Subjects were initially identified from two NHANES cycles: 2009–2010 (n=10,537) and 2011–2012 (n=9,756), totalling 20,293 participants. Subjects with available oral microbiome data (n=9,847, with 1,325 taxa) were first selected. Sequential exclusion criteria were applied, with subjects with fewer than 5,000 microbiome reads were excluded (n = 293); subjects younger than 18 years old (n = 1,131) and those who were pregnant (n = 100) were excluded; and subjects lacking dentition records (n = 1,127), having only periodontal or only caries records (n = 3,424), or having only one remaining tooth (n = 2), which precluded periodontal assessment, were excluded.

After these steps, 3,770 participants remained with complete oral health records and high-quality oral microbiome data. Participants were classified into five oral condition categories: oral health, caries only, periodontitis only, caries + periodontitis, as well as edentulous status. Microbial taxa were further filtered by excluding: taxa with ≤10 reads in more than 70% of individuals across all oral conditions (n = 1,246); and taxa lacking microbiome class information or known biological annotations (n = 2). The final analytical dataset included 3,770 participants and 77 microbial taxa used for further microbial analyses.

(B) Availability of oral health records based on clinical periodontal and dental examination among NHANES 2009-2011 subjects.

(C) Classification of oral conditions based on caries and periodontitis status according to the CDC/AAP and ACES periodontitis definitions.


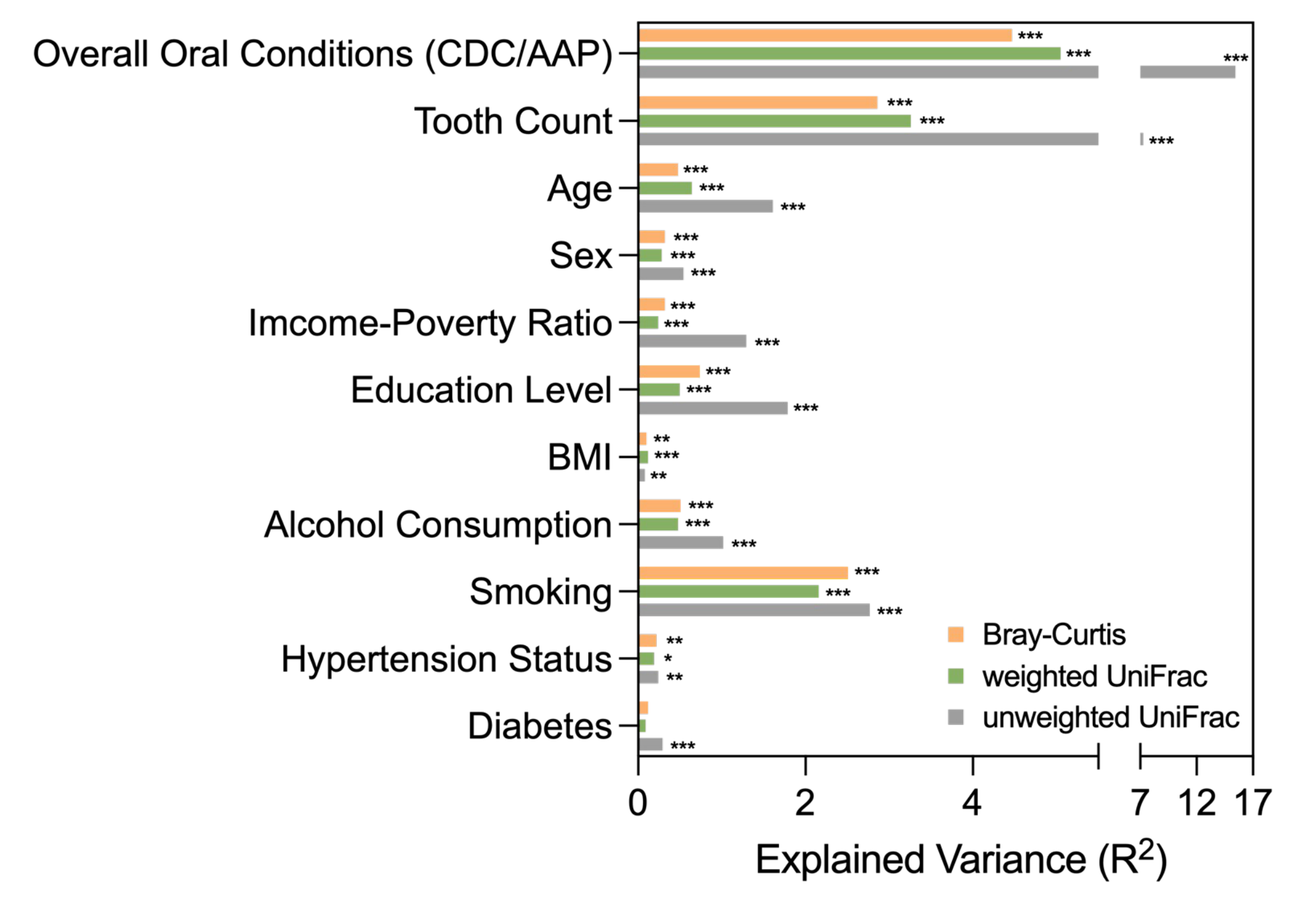


**Figure S2 Explained variance of microbial community based on non-compositional analysis.**

Variance of microbial community based on the Bray-Curtis dissimilarities, weighted UniFrac distances, and unweighted UniFrac distances from read count data, explained by oral conditions, sociodemographic factors, and general health conditions. R² and *p* values were calculated by PERMANOVA test. Significance levels are denoted as *: *p* <0.05, **: *p* <0.01, ***: *p* <0.001.


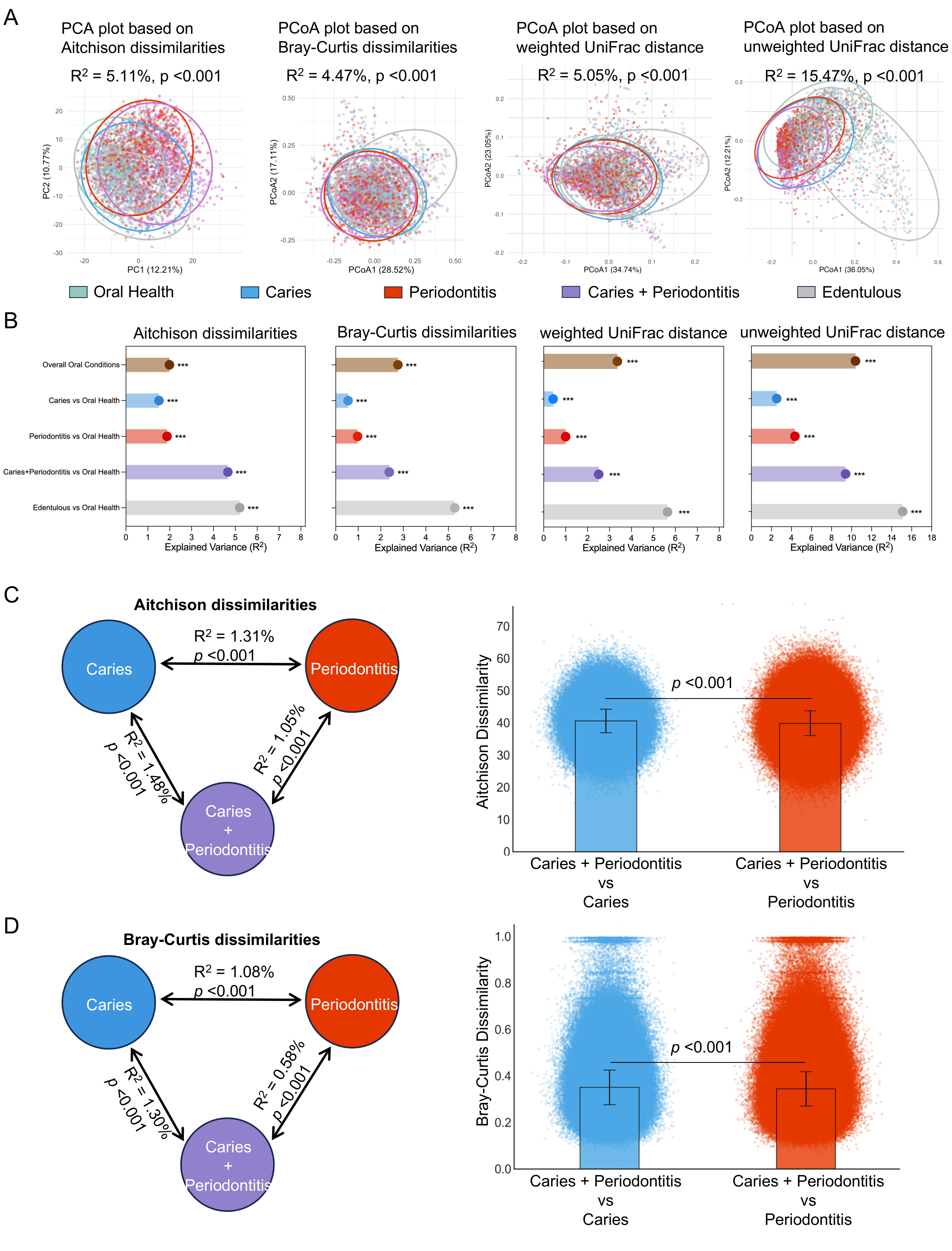


**Figure S3.** **Variation in oral microbiota composition explained by specific oral conditions using different data analysis approaches, with oral conditions defined based on CDC/AAP periodontitis definition**

(A) Visualization of dissimilarities in microbial community of different oral conditions. ; Principal component analysis (PCA) was used for Aitchison dissimilarities based on CLR transformed data; Principal coordinate analysis (PCoA) were used for Bray-Curtis dissimilarities, weighted UniFrac distances, unweighted UniFrac distances based on read count data.Each point represents an individual; ellipses indicate 95% confidence regions. Explained variance (R²) and p-values calculated by PERMANOVA test;

(B) Variation explained by each oral conditions defined by caries, periodontitis and dentition status, R² and p-values were calculated on by PERMANOVA test, based on Aitchison dissimilarities for CLR transformed data, and Bray-Curtis dissimilarities, weighted UniFrac distances, unweighted UniFrac distances for read count data respectively;

(C) Inter-group dissimilarities based on Aitchison dissimilarities on CLR-transformed data. Explained variance among the three disease groups (caries, periodontitis, and caries+periodontitis) were calculated PERMANOVA test; Pairwise comparison of dissimilarities between the co-existing disease group and each single disease group (caries+periodontitis vs. caries, and caries+periodontitis vs. periodontitis) was calculated by Mann-Whitney U test.

(D) Inter-group dissimilarities based on Bray-Curtis dissimilarities. Explained variance among the three disease groups (caries, periodontitis, and caries+periodontitis) were calculated PERMANOVA test; Pairwise comparison of dissimilarities between the co-existing disease group and each single disease group (caries+periodontitis vs. caries, and caries+periodontitis vs. periodontitis) was calculated by Mann-Whitney U test.

Periodontitis status is defined based on the CDC/AAP definition.


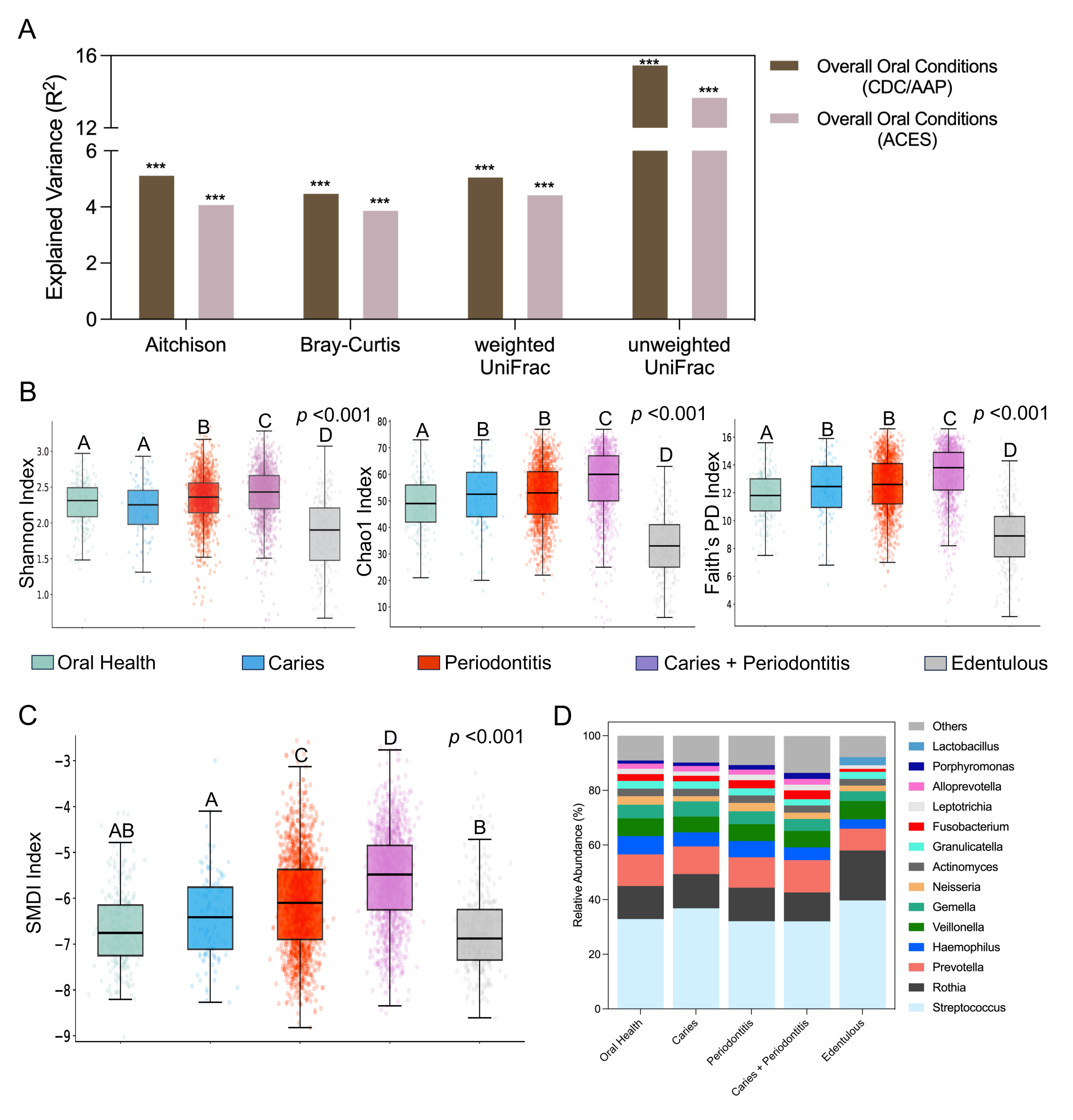


**Figure S4 Association between oral conditions and oral microbiome based on ACES periodontitis definition**

(A) Variance of microbial community based on the Aitchison dissimilarities from CLR transformed data, and Bray-Curtis dissimilarities, weighted UniFrac distances, unweighted UniFrac distances from the read count data, explained by oral health status based on CDC/AAP and ACES periodontitis definitions. R² and *p* values were calculated by PERMANOVA test; (B) α-diversity comparisons across five oral conditions, measured by Shannon, Chao1, and Faith’s PD indices; (C) Changes in periodontitis-related microbiome dysbiosis across five oral conditions, measured by the subgingival microbial dysbiosis index (SMDI); (D) Average relative abundances of the most dominant taxa (>1% average relative abundance) in different oral conditions. In (A), significance levels are denoted as *: *p* <0.05, **: *p* <0.01, ***: *p* <0.001. For (B) and (C), the *p* value represents the results of the Kruskal-Wallis test across all groups. Post-hoc pairwise comparisons were conducted using Dunn’s test, with different letters above bars representing statistical differences (*p* <0.05), while identical letters indicate no significant difference. Periodontitis status is defined based on the ACES definition. The comparison of α-diversity and SMDI across different oral conditions accounted for the NHANES complex survey design.


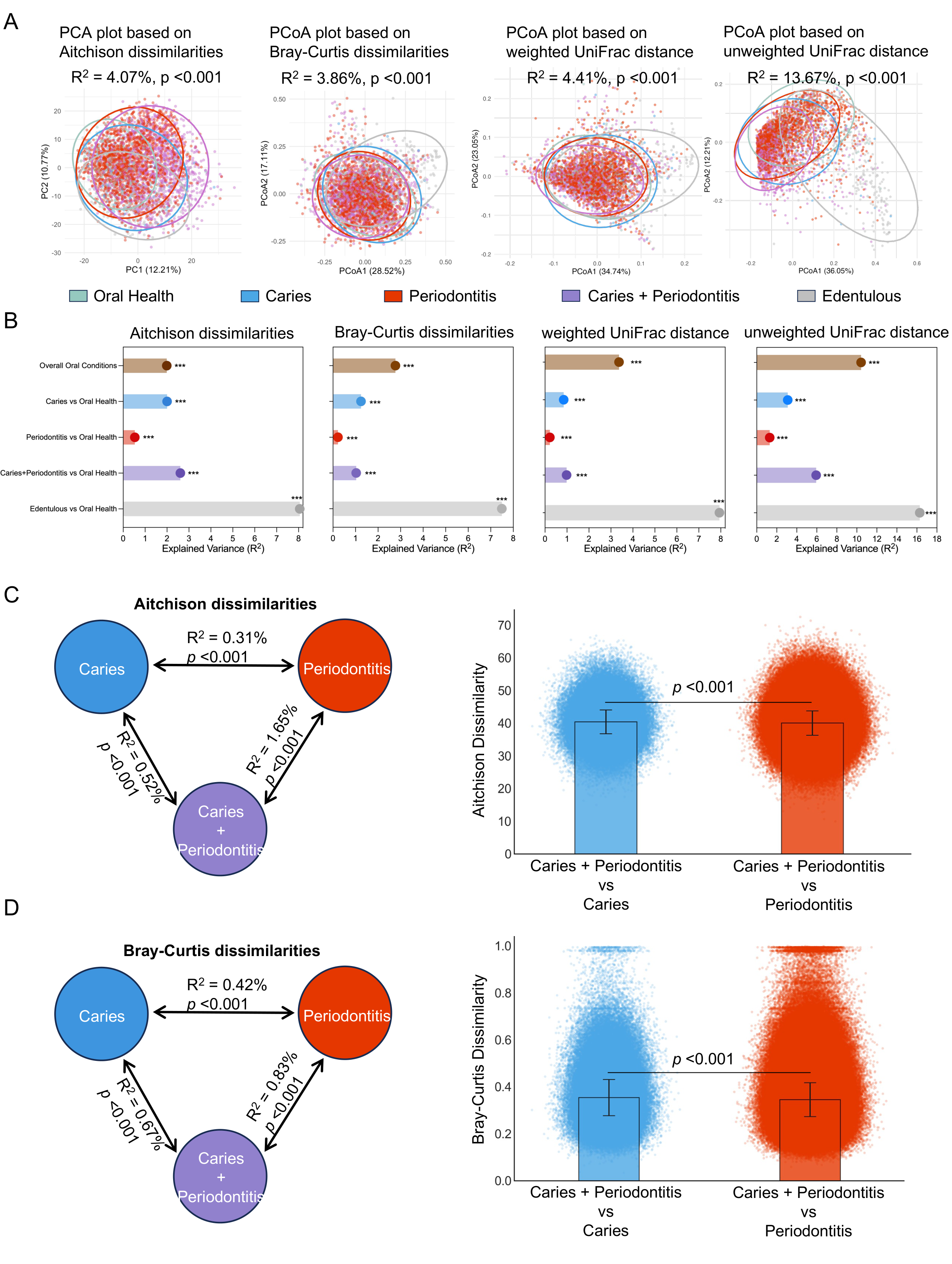


**Figure S5. Variation in oral microbiota composition explained by specific oral conditions using different data analysis approaches, with oral conditions defined based on ACES periodontitis definition**

(A) Visualization of dissimilarities in microbial community of different oral conditions. Principal component analysis (PCA) was used for Aitchison dissimilarities based on CLR transformed data; Principal coordinate analysis (PCoA) were used for Bray-Curtis dissimilarities, weighted UniFrac distances, unweighted UniFrac distances based on read count data. Each point represents an individual; ellipses indicate 95% confidence regions. Explained variance (R²) and p-values calculated by PERMANOVA test;

(B) Variation explained by each oral conditions defined by caries, periodontitis and dentition status, R² and p-values were calculated on by PERMANOVA test, based on Aitchison dissimilarities for CLR transformed data, and Bray-Curtis dissimilarities, weighted UniFrac distances, unweighted UniFrac distances for read count data respectively;

(C) Inter-group dissimilarities based on Aitchison dissimilarities on CLR-transformed data. Explained variance among the three disease groups (caries, periodontitis, and caries+periodontitis) were calculated PERMANOVA test; Pairwise comparison of dissimilarities between the co-existing disease group and each single disease group (caries+periodontitis vs. caries, and caries+periodontitis vs. periodontitis) was calculated by Mann-Whitney U test.

(D) Inter-group dissimilarities based on Bray-Curtis dissimilarities. Explained variance among the three disease groups (caries, periodontitis, and caries+periodontitis) were calculated PERMANOVA test; Pairwise comparison of dissimilarities between the co-existing disease group and each single disease group (caries+periodontitis vs. caries, and caries+periodontitis vs. periodontitis) was calculated by Mann-Whitney U test.

Periodontitis status is defined based on the ACES definition.


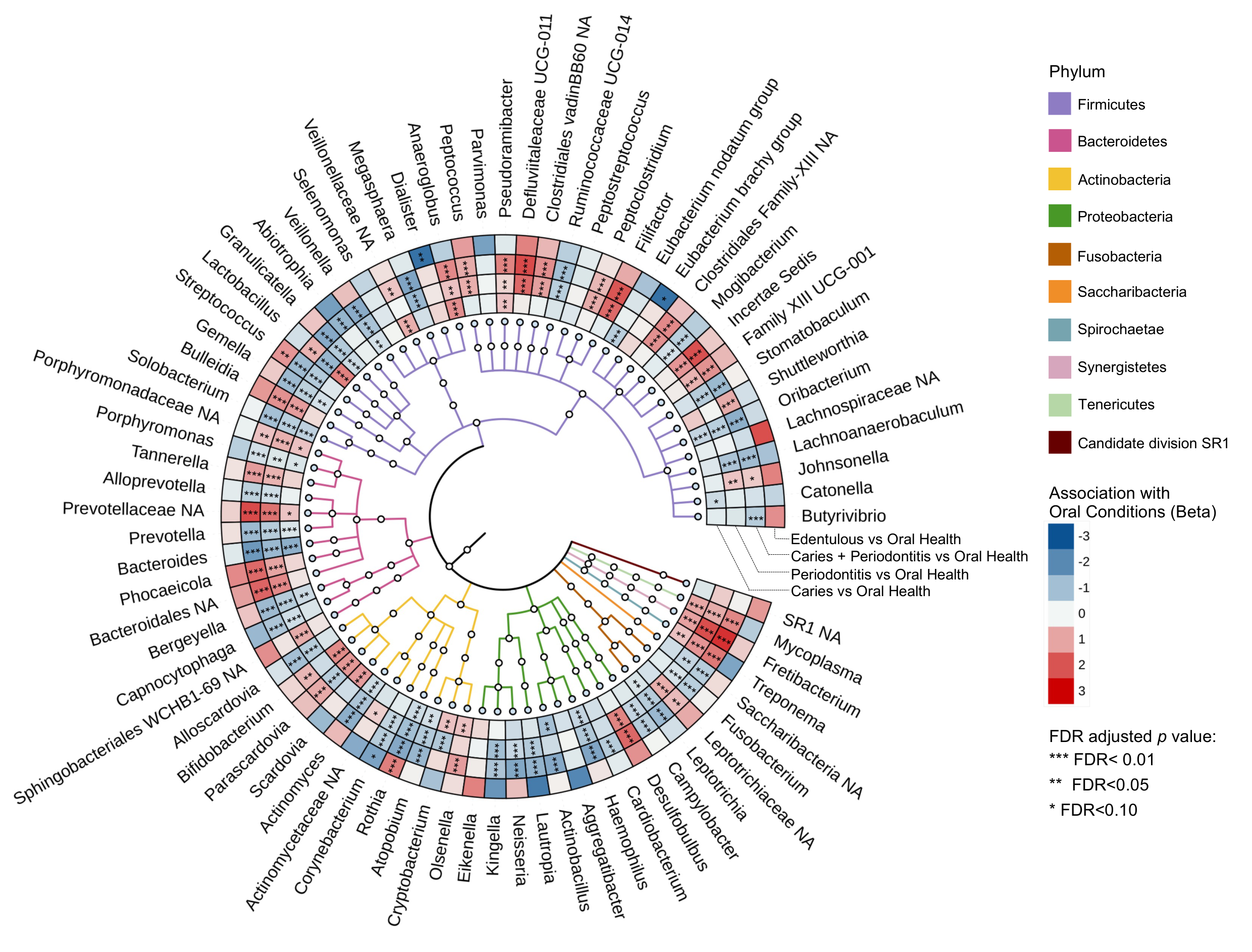


**Figure S6 Association between oral rinse microbiota and oral conditions visualized by** **phylogenetic tree, with oral conditions defined by CDC/AAP periodontitis definition, and additionally adjusted for age, smoking status and tooth numbers**

The diagram illustrates the taxa positively associated (blue boxes in the outer wheels) and negatively associated (red boxes) in relation to oral health compared to edentulism, caries, periodontitis, or the co-occurrence of caries and periodontitis within microbial phylogenetic trees. In the heatmap, color intensity indicates the strength and direction of associations between taxa and oral conditions (each compared to oral health). Asterisks within each box indicate the significance of associations, as *: *p* <0.05, **: *p* <0.01, ***: *p* <0.001. The associations were evaluated by MaAsLin2 approach, based on CLR-transformed data, and adjusted for sex, race/ethnicity, body mass index, income-to-poverty ratio, education level, diabetes, hypertension status, age, smoking status, and tooth numbers. *p* values have been adjusted for multiple comparisons using the false discovery rate method (FDR) with a target rate of 0.1. Periodontitis status is defined based on the CDC/AAP definition. Colors on the phylogenetic tree's clade denote different bacterial phyla.


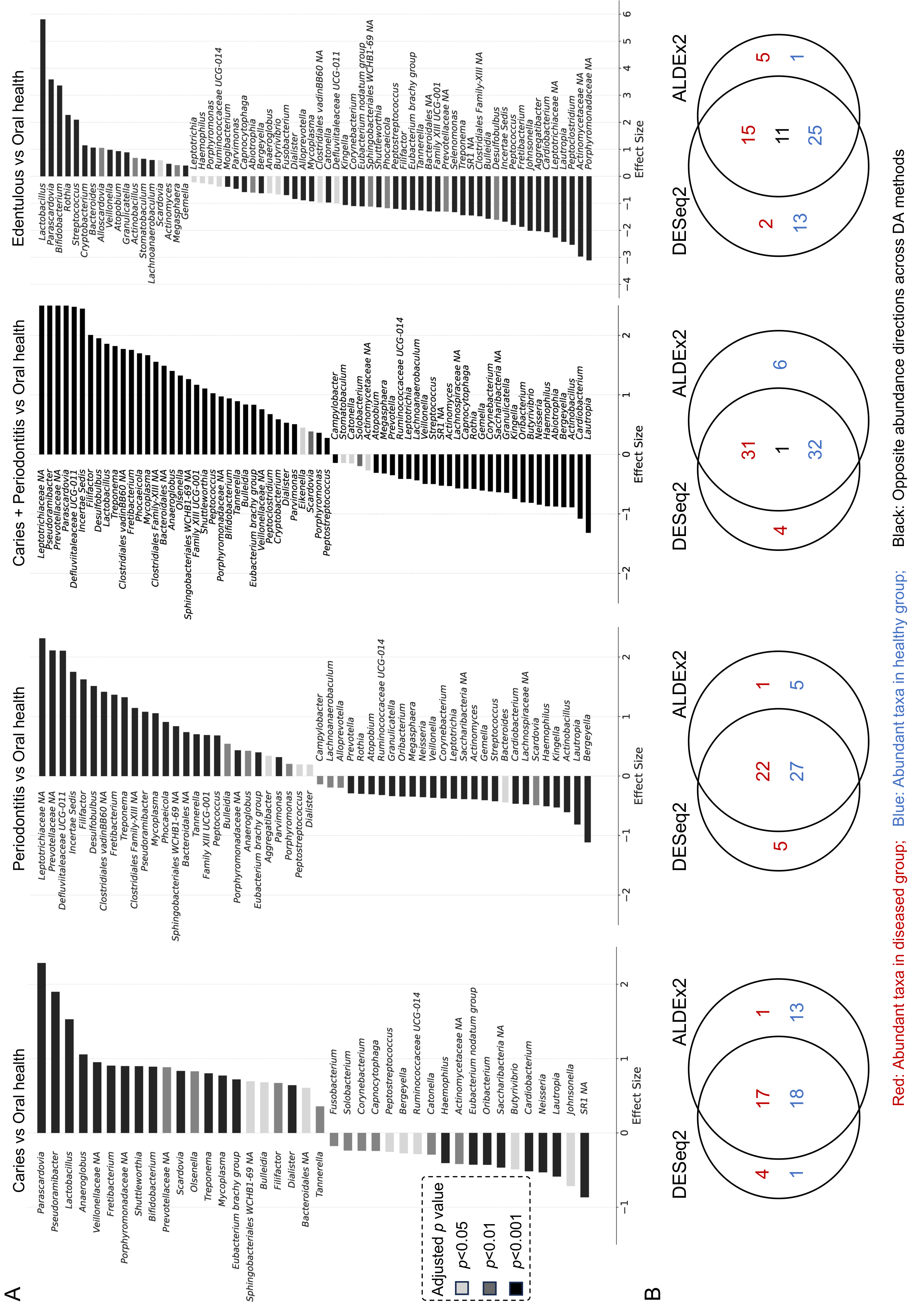


**Figure S7 Differential abundance of taxa across oral healthy and diseased groups identified using DESeq2, with oral conditions defined based on CDC/AAP periodontitis definition** (A) Bar plots displaying differential abundance comparisons between each oral conditions and oral health group by DESeq2. Taxa with adjusted *p* value <0.05 are shown in the plot. Analyses were performed on read count data, Log₂ fold changes estimated by the DESeq2 model were directly used for visualization in volcano and bar plots. Analyses were adjusted by false discovery rate (FDR) for multiple testing. Oral conditions compared to oral health included caries-only, periodontitis-only, co-existing caries and periodontitis, and edentulous status. Periodontitis status is defined based on the CDC/AAP definition. Detailed DESeq2 results are provided in Supplementary Spreadsheet 2.

(B) Venn plots comparing significant differential abundance results obtained from DESeq2 and ALDEx2, under identical oral condition contrasts. Red numbers indicate the abundant taxa in diseased group, blue numbers indicate the abundant taxa in healthy group, while black numbers indicate taxa with opposite direction of differential abundance between DESeq2 and ALDEx2. The corresponding taxa lists and differential abundance results are provided in Supplementary Spreadsheet 4. DA: differential abundance.


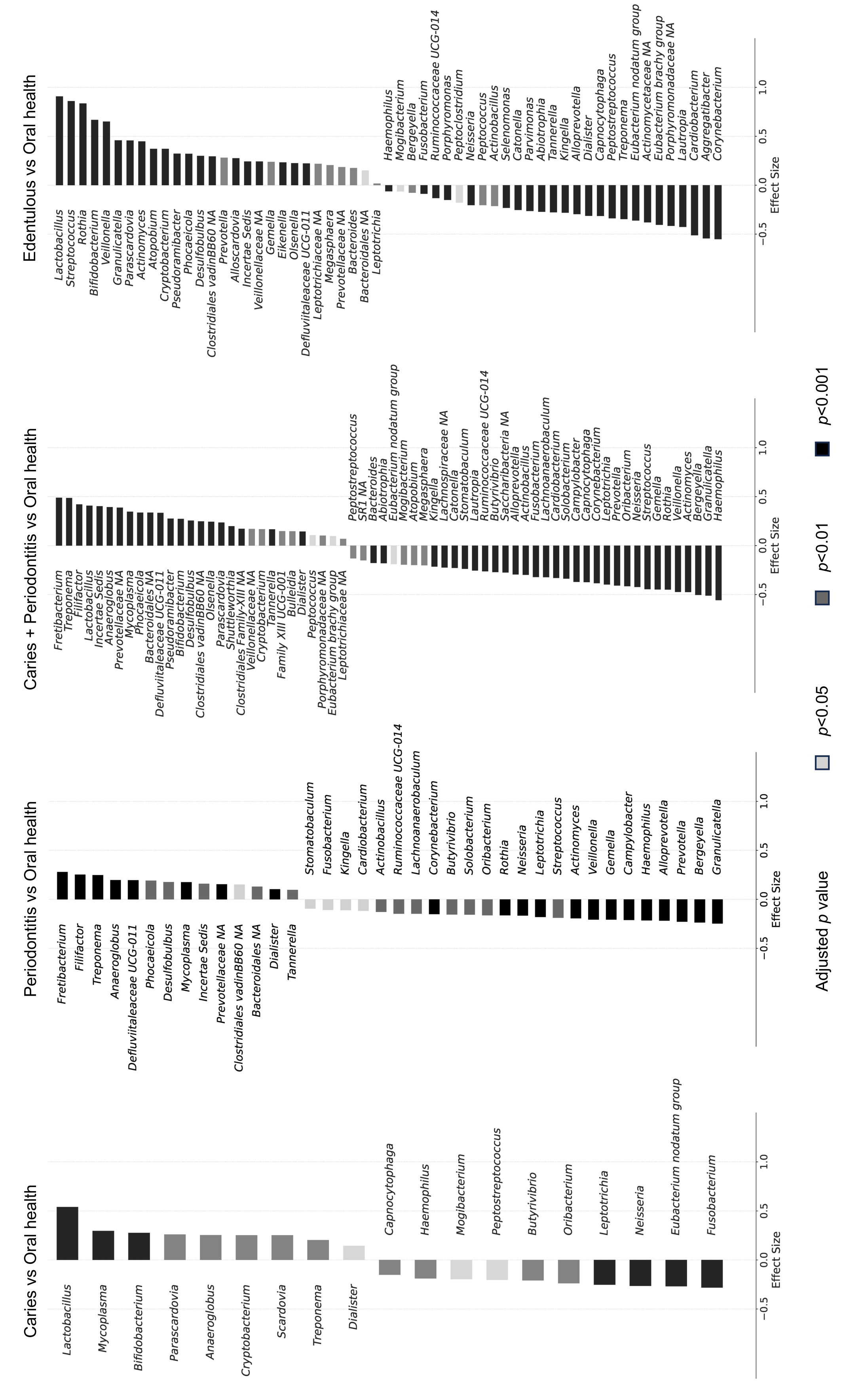


**Figure S8 Differential abundance of taxa across oral healthy and diseased groups identified using ALDEx2, with oral conditions defined based on ACES periodontitis definition**

Bar plots displaying differential abundance comparisons between each oral conditions and oral health group by ALDEx2. Taxa with adjusted p value <0.05 are shown in the plot. Analyses were performed with a centered log-ratio (CLR) transformation of the count data with Monte Carlo sampling from the Dirichlet distribution, the estimated effect size was directly used for visualization, representing the between-group difference in CLR abundance. Analyses were adjusted by false discovery rate (FDR) for multiple testing. Oral conditions compared to oral health included caries-only, periodontitis-only, co-existing caries and periodontitis, and edentulous status. Periodontitis status is defined based on the ACES definition. Detailed ALDEx2 results are provided in Supplementary Spreadsheet 1.


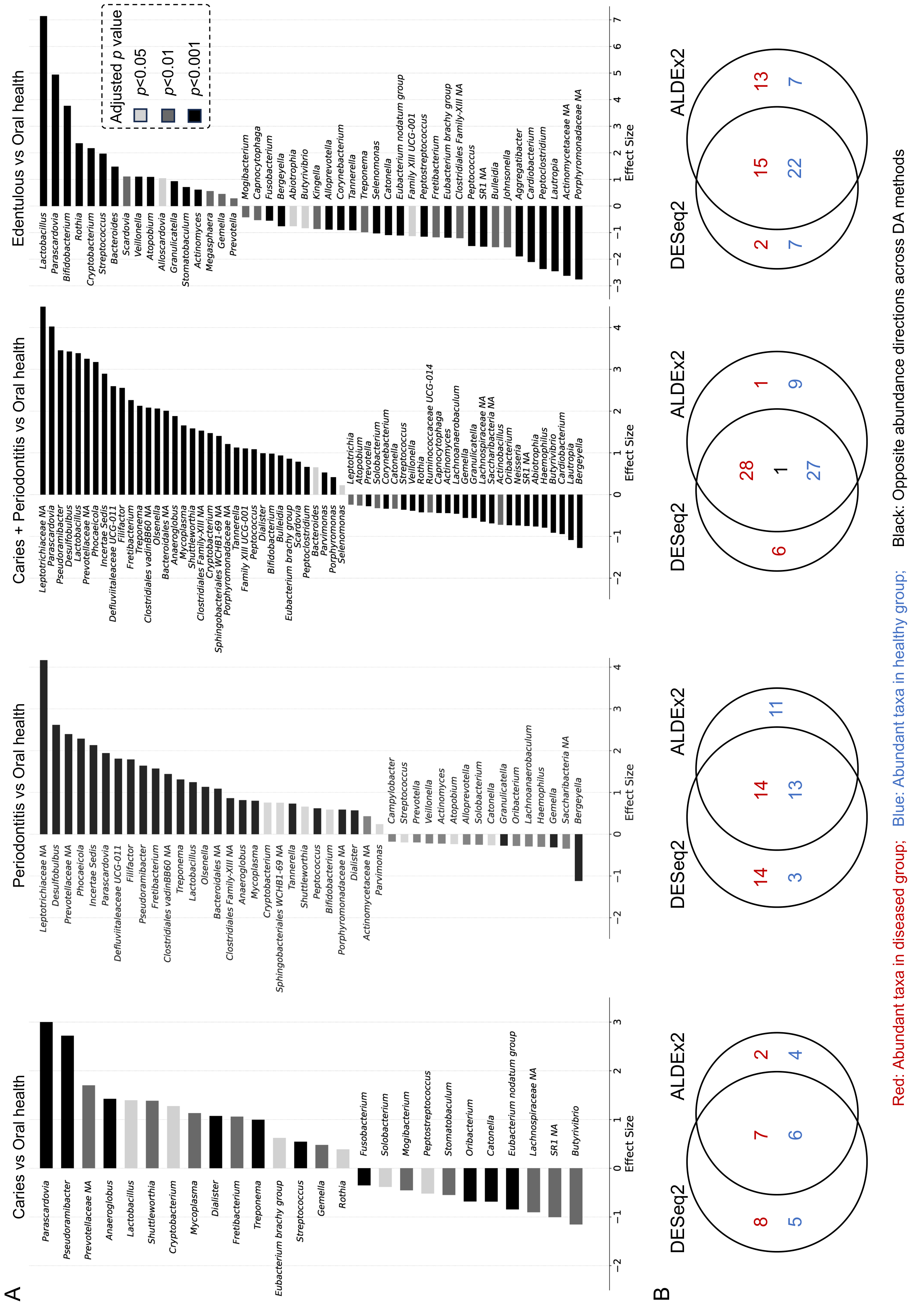


**Figure S9 Differential abundance of taxa across oral healthy and diseased groups identified using DESeq2, with oral conditions defined based on ACES periodontitis definition**

(A) Bar plots displaying differential abundance comparisons between each oral conditions and oral health group by DESeq2. Taxa with adjusted *p* value <0.05 are shown in the plot. Analyses were performed on read count data, Log₂ fold changes estimated by the DESeq2 model were directly used for visualization in volcano and bar plots. Analyses were adjusted by false discovery rate (FDR) for multiple testing. Oral conditions compared to oral health included caries-only, periodontitis-only, co-existing caries and periodontitis, and edentulous status. Periodontitis status is defined based on the ACES definition. Detailed DESeq2 results are provided in Supplementary Spreadsheet 2.

(B) Venn plots comparing significant differential abundance results obtained from DESeq2 and ALDEx2, under identical oral condition contrasts. Red numbers indicate the abundant taxa in diseased group, blue numbers indicate the abundant taxa in healthy group, while black numbers indicate taxa with opposite direction of differential abundance between DESeq2 and ALDEx2. The corresponding taxa lists and differential abundance results are provided in Supplementary Spreadsheet 4. DA: differential abundance.


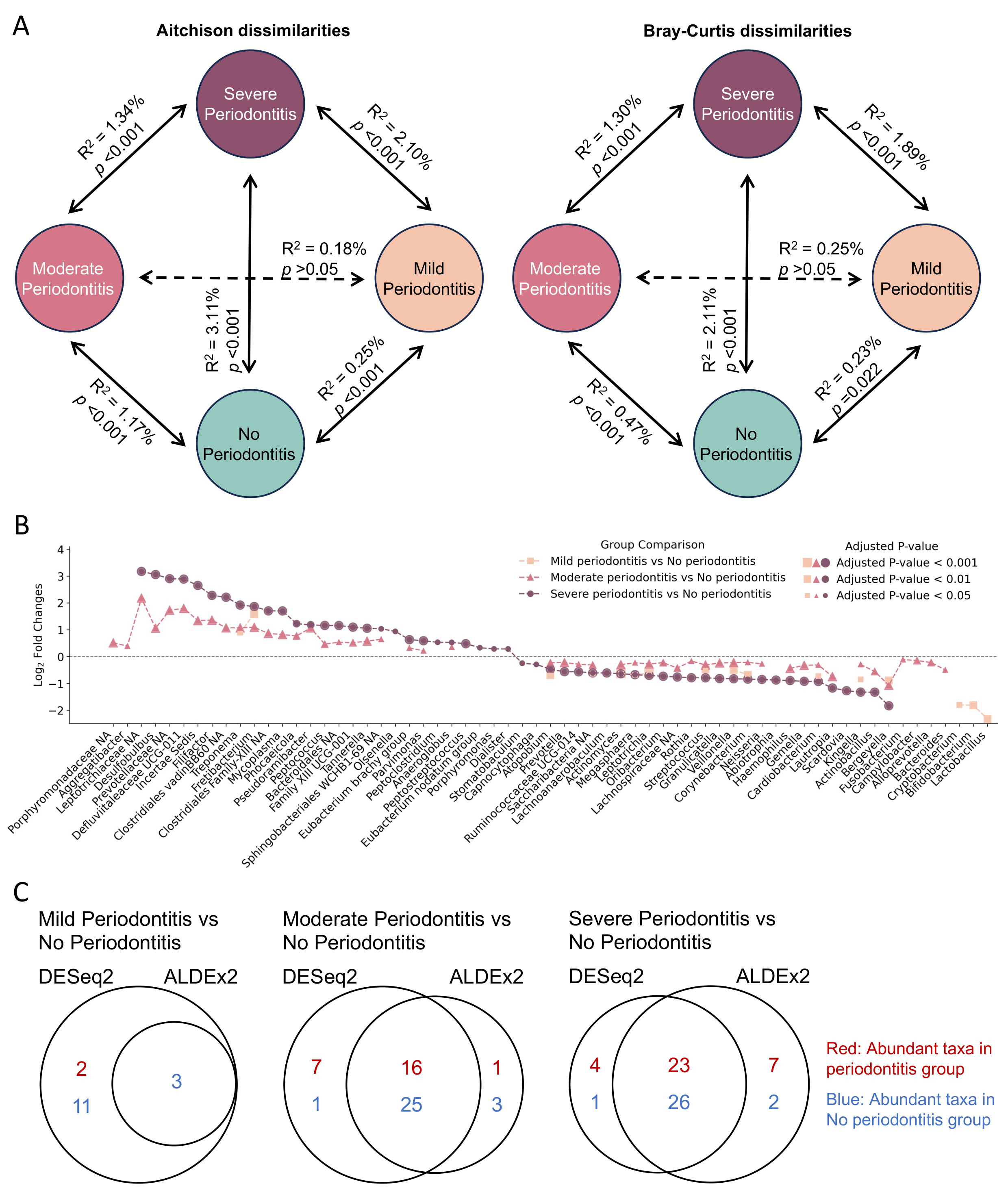


**Figure S10 Dose-dependent association between oral microbiome profiles and periodontitis severity, with oral conditions defined based on CDC/AAP periodontitis definition**

(A) Explained variance (R²) among the four periodontitis severity groups as defined by CDC/AAP definition (no periodontitis, mild periodontitis, moderate periodontitis, severe periodontitis), calculated based on Aitchison dissimilarities with CLR-transformed data and Bray-Curtis dissimilarities with read count data by PERMANOVA test.

(B) Differential abundance comparisons between relatively healthy periodontal status (no periodontitis under CDC/AAP) and more advanced stages of periodontitis. Each dot represents a taxon with significant differences, the dot position reflects the magnitude of change (log₂-transformed fold change), and the dot size indicates the level of statistical significance (adjusted *p*-value). Analyses were performed using DESeq2 on read count data, followed by false discovery rate (FDR) correction. Taxa with adjusted *p* value <0.05 underwent log₂ transformation for visualization..

(C) Venn plots comparing significant differential abundance results obtained from DESeq2 and ALDEx2, under identical periodontitis status contrasts. Red numbers indicate the abundant taxa in diseased group, and blue numbers indicate the abundant taxa in healthy group. The corresponding taxa lists and differential abundance results are provided in Supplementary Spreadsheet 5.


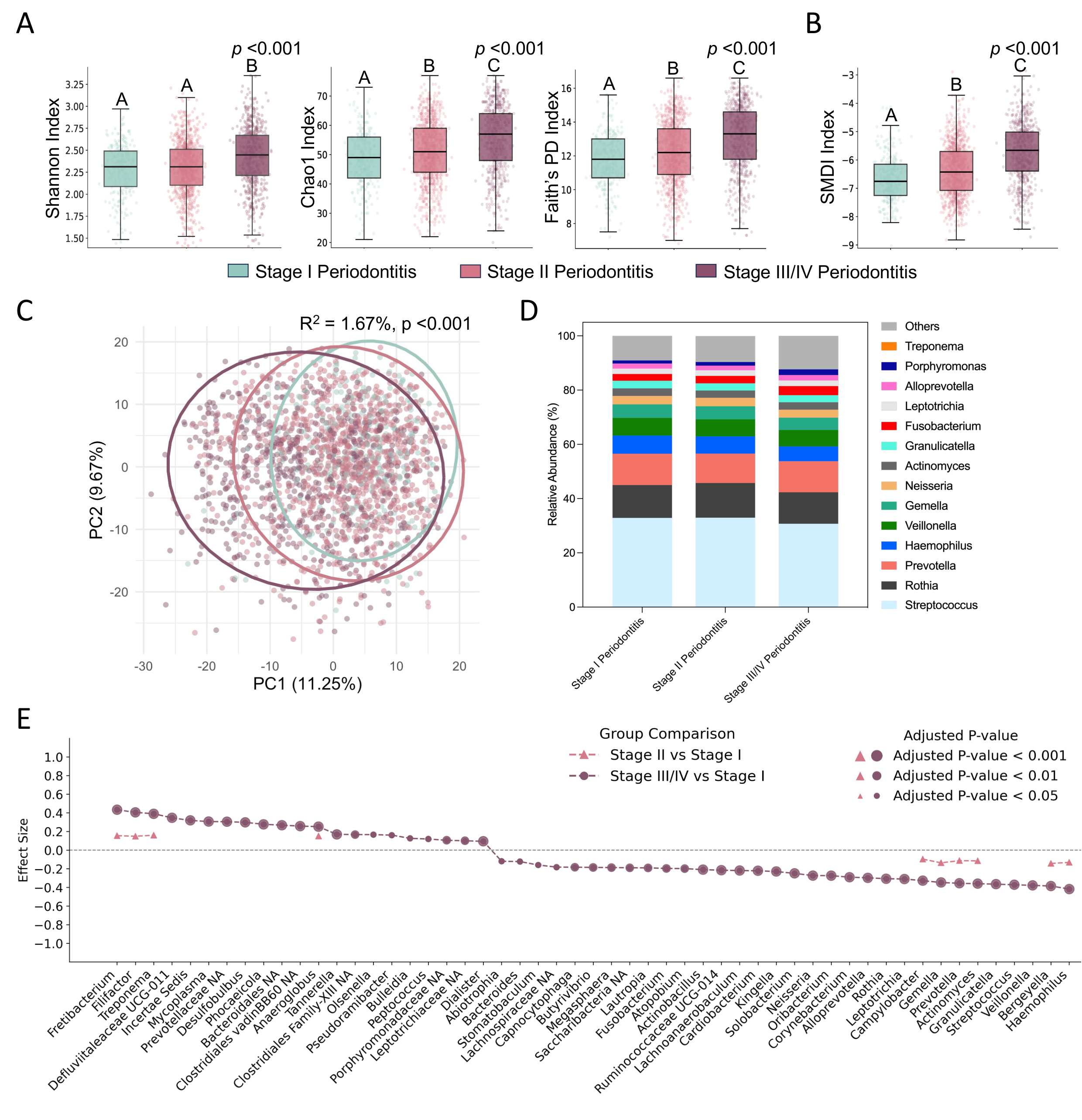


**Figure S11 Dose-dependent association between oral microbiome profiles and periodontitis severity under the ACES definition**

(A) α-diversity comparisons across periodontitis severity status, measured by Shannon, Chao1 and Faith’s PD indices;

(B) Changes in periodontitis-related microbiome dysbiosis across periodontitis severity status, measured by the subgingival microbial dysbiosis index (SMDI);

(C) Principal component analysis (PCA) based on Aitchison dissimilarities of CLR transformed data, comparing microbial community structures across periodontitis severity status. Each point represents an individual; ellipses indicate 95% confidence regions. Explained variance (R²) and *p* values were calculated by PERMANOVA test;

(D) Average relative abundances of the most dominant taxa (>1% average relative abundance) across periodontitis severity status;

(E) Differential abundance comparisons between relatively healthy periodontal status (stage I periodontitis in ACES definition) and more advanced periodontitis stages. Each dot represents a taxon with significant changes, with dot position indicating the magnitude of change (effect size estimated from between-group CLR differences) and dot size reflecting adjusted *p* value significance. Statistical significance was assessed using Welch’s t-test and Wilcoxon rank test on Monte Carlo samples generated by ALDEx2 on CLR-transformed data, followed by false discovery rate (FDR) correction for multiple testing. Only taxa with adjusted p-values < 0.05 are shown in the plot. For (A) and (B), *p* values represent Kruskal-Wallis test results across all groups. Post-hoc pairwise comparisons were conducted using Dunn’s test; different letters above bars indicate statistically significant differences (*p* < 0.05), while identical letters indicate no significant difference. Periodontitis status is defined based on the ACES definition. The comparison of α-diversity and SMDI across different oral conditions accounted for the NHANES complex survey design.


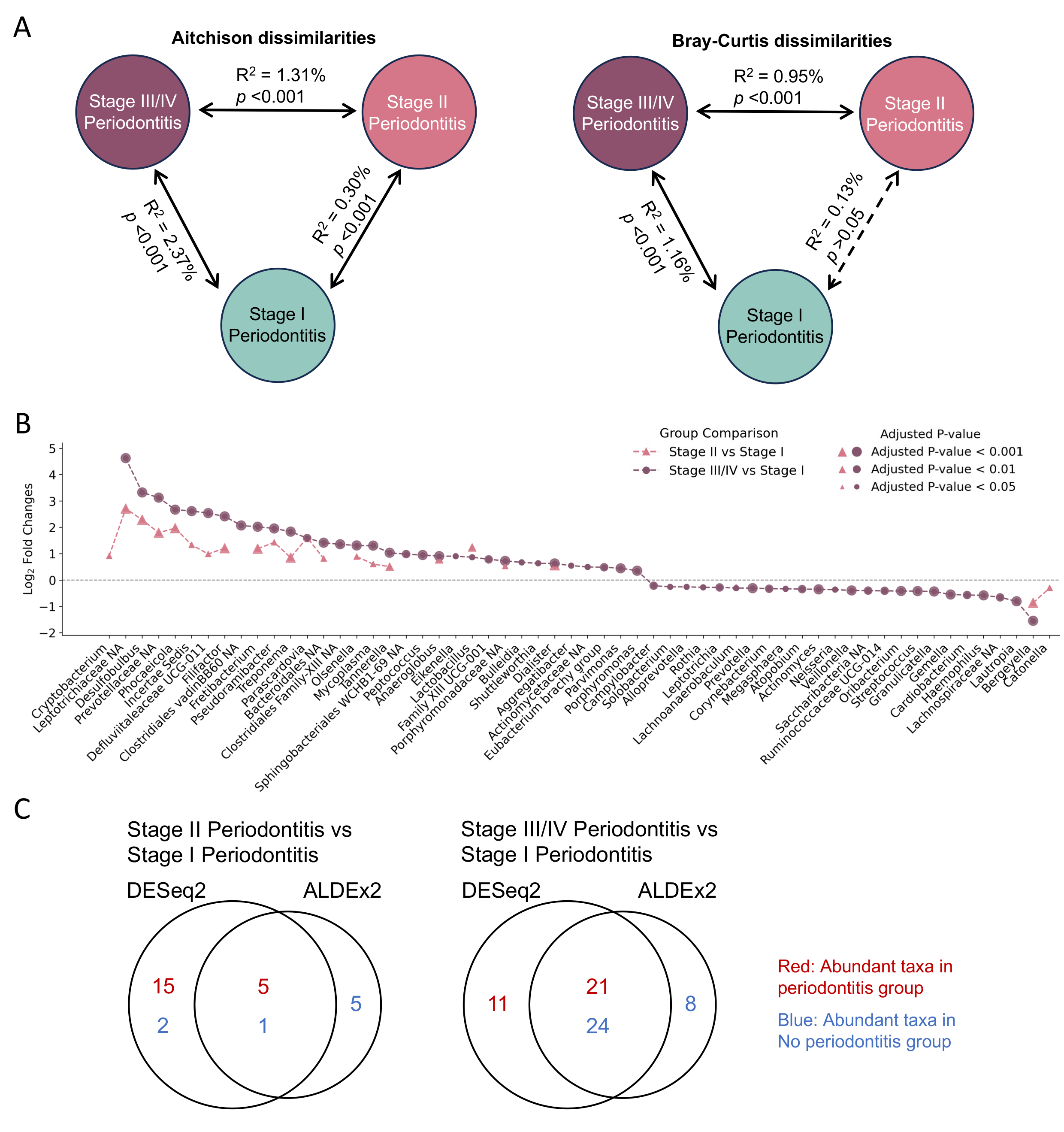


**Figure S12 Dose-dependent association between oral microbiome profiles and periodontitis severity, with oral conditions defined based on ACES periodontitis definition**

(A) Explained variance (R²) among the four periodontitis severity groups as defined by ACES definition (stage I periodontitis, stage II periodontitis, stage III/IV periodontitis), calculated based on Aitchison dissimilarities with CLR-transformed data and Bray-Curtis dissimilarities with read count data by PERMANOVA test.

(B) Differential abundance comparisons between relatively healthy periodontal status (Stage I periodontitis under ACES) and more advanced stages of periodontitis. Each dot represents a taxon with significant differences, the dot position reflects the magnitude of change (log₂-transformed fold change), and the dot size indicates the level of statistical significance (adjusted *p*-value). Analyses were performed using DESeq2 on read count data, followed by false discovery rate (FDR) correction. Taxa with adjusted *p* value <0.05 underwent log₂ transformation for visualization.

(C) Venn plots comparing significant differential abundance results obtained from DESeq2 and ALDEx2, under identical periodontitis status contrasts. Red numbers indicate the abundant taxa in diseased group, and blue numbers indicate the abundant taxa in healthy group. The corresponding taxa lists and differential abundance results are provided in Supplementary Spreadsheet 5.


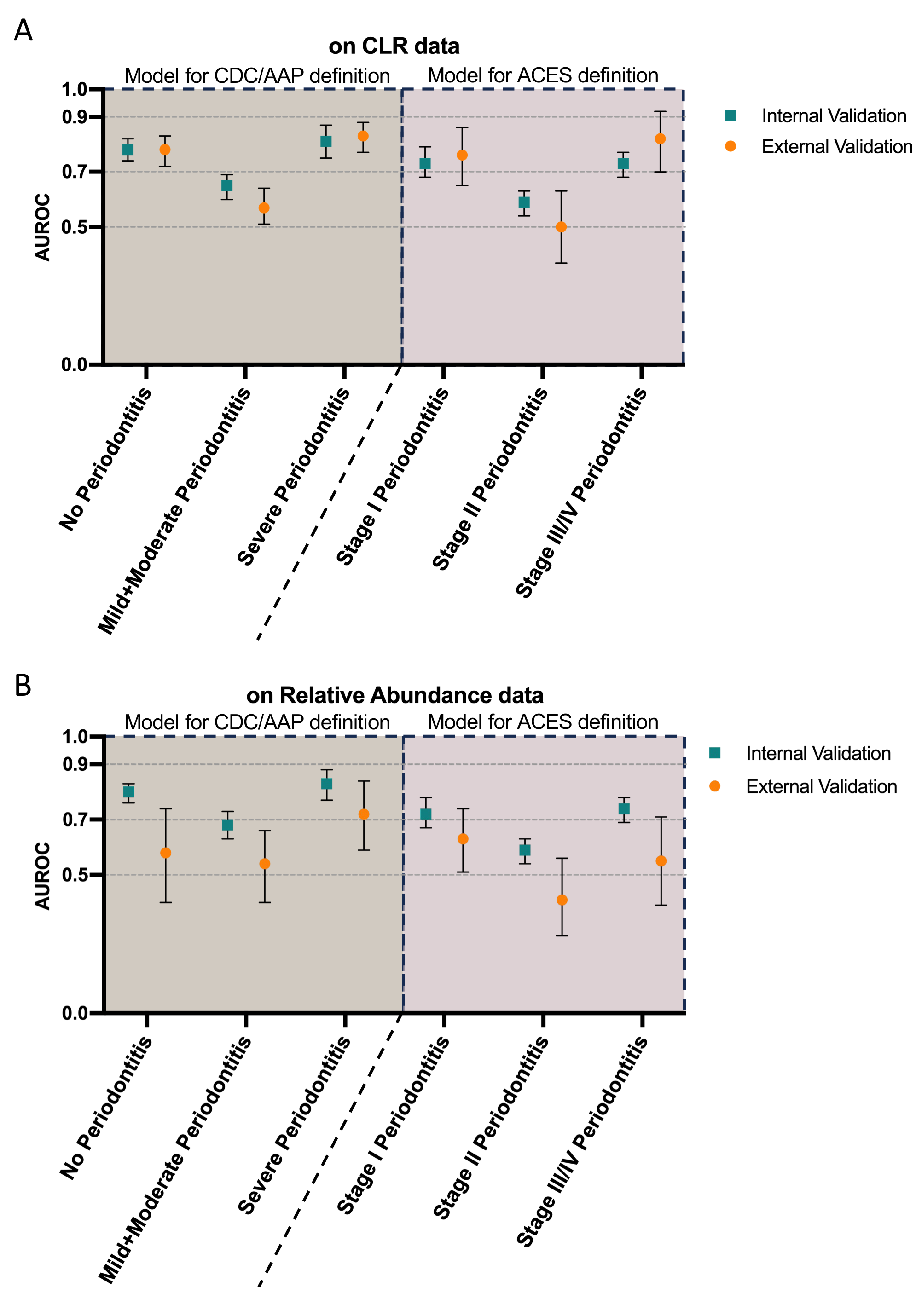


**Figure S13 Comparison of machine learning model performance between internal and external validation.**

(A) Comparison of AUROC between internal and external validation for the optimal random forest model developed on CLR-transformed data from the NHANES database, using either the CDC/AAP or ACES definition of periodontitis. Internal validation was conducted with the independent held-out test set within NHANES, and external validation was performed using a local cohort established by the researchers.

(B) Comparison of AUROC between internal and external validation for the optimal random forest model developed on relative abundance data from the NHANES database, using either the CDC/AAP or ACES definition of periodontitis. Internal validation was conducted with the independent held-out test set within NHANES, and external validation was performed using a local cohort established by the researchers.


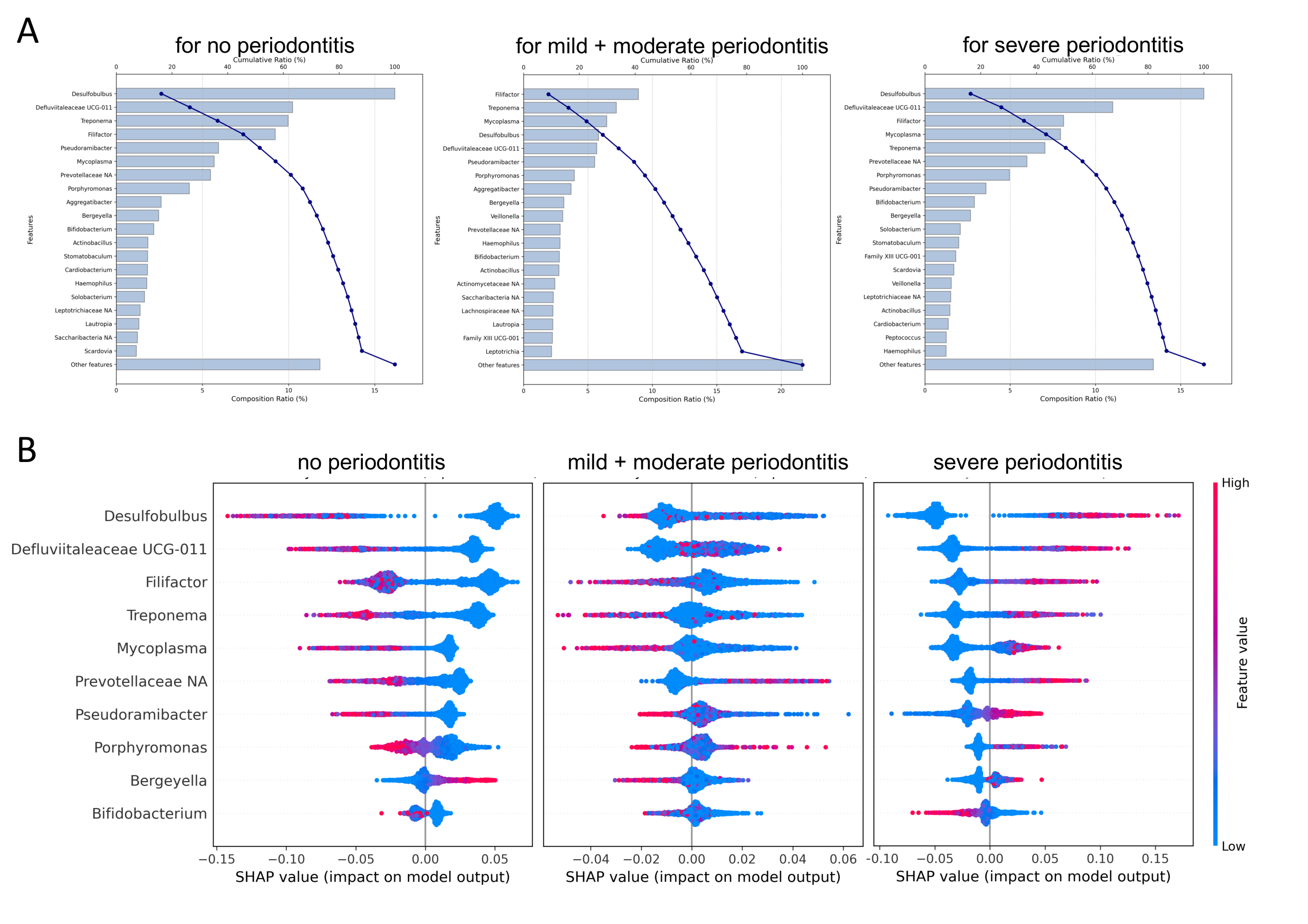


**Figure S14 Periodontitis-associated taxa identified by SHAP in relative abundance data based models across different periodontitis severity status defined by the CDC/AAP definition.**

(A) Pareto plots showing the single and cumulative contribution of dominant taxa to model interpretability, as determined by the random forest multi-class model;

(B) Shapley additive explanation (SHAP) summary plots for the top10 taxa contributing to model interpretability. Each point represents a sample, colored by the relative abundance of the corresponding taxon (blue to red representing low to high abundance). The x-axis shows the SHAP value, indicating both the magnitude and direction of each taxon’s impact on the model output.


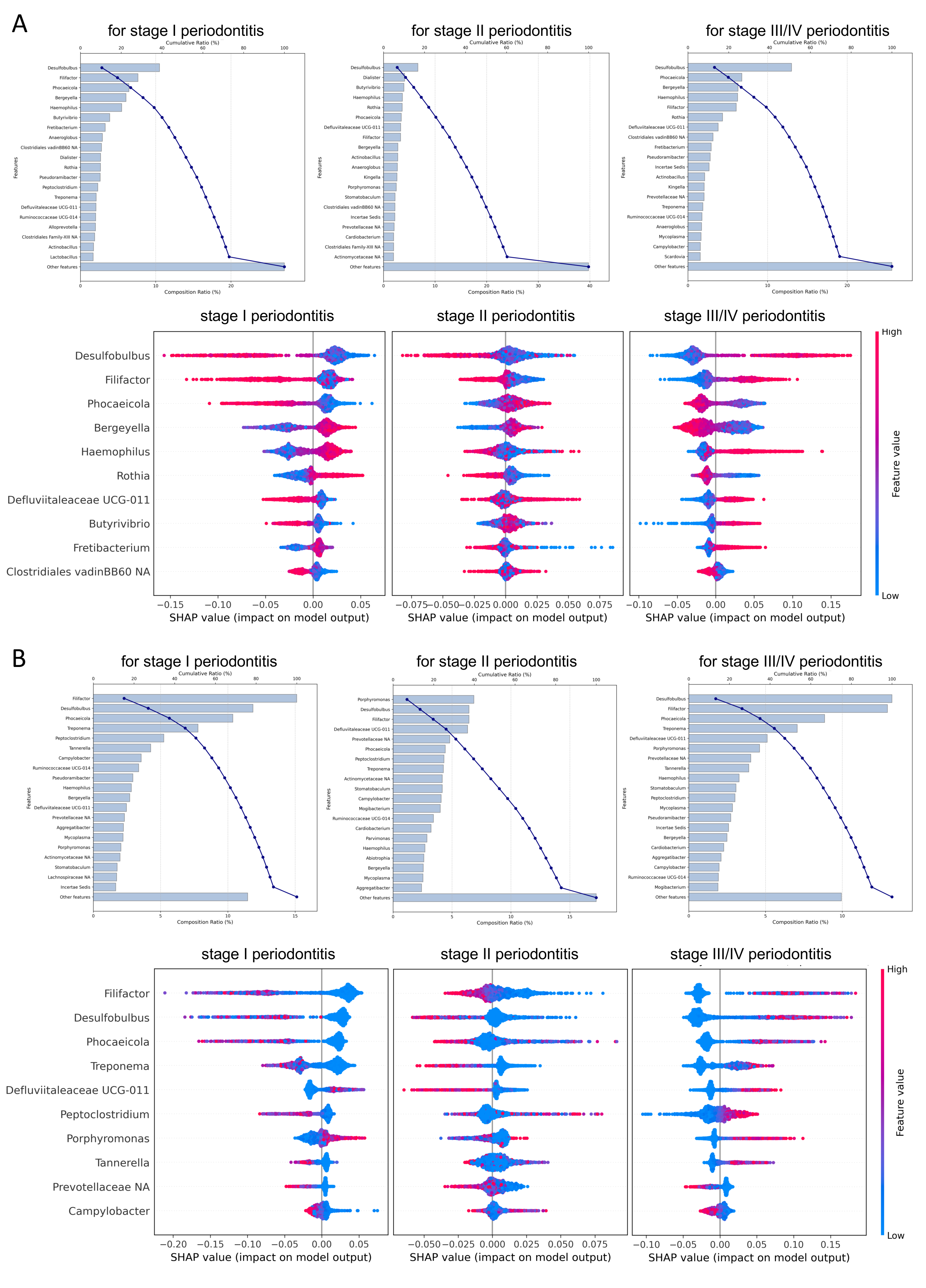


**Figure S15 Periodontitis-associated taxa identified by SHAP in CLR-transformed data and relative abundance data based models across different periodontitis severity status defined by the ACES definition.**

(A) Pareto plots and SHAP summary plots for the top-contributing taxa in the CLR-transformed data based random forest multi-class model;

(B) Pareto plots and SHAP summary plots for the top-contributing taxa in the relative abundance data based random forest multi-class model;

Pareto plots showing the single and cumulative contribution of dominant taxa to model interpretability; SHAP summary plots for the top10 taxa contributing to model interpretability. Each point represents a sample, colored by the CLR value/relative abundance of the corresponding taxon (blue to red representing low to high CLR value/relative abundance). The x-axis shows the SHAP value, indicating both the magnitude and direction of each taxon’s impact on the model output.

**Supplementary Tables**

**Table S1 Characteristics of study participants with different oral health conditions based on caries and periodontitis status with ACES periodontitis definitions.**

| **ACES definition** | **Overall** | **Edentulous** | **Oral Health** | **Caries** | **Periodontitis** | **Caries+Periodontitis** | ***p* value** |
| --- | --- | --- | --- | --- | --- | --- | --- |
| **N** | 3770 | 293 | 264 | 118 | 1669 | 1426 |  |
| Age | 49 (39-59) | 61 (54-65) | 40 (33-48) | 38 (34-44) | 50 (40-60) | 49 (40-58) | **<0.001** |
| Sex |  |  |  |  |  |  | **<0.001** |
| Male | 1955 (51.9%) | 154 (52.6%) | 82 (31.1%) | 39 (33.1%) | 836 (50.1%) | 844 (59.2%) |  |
| Female | 1815 (48.1%) | 139 (47.4%) | 182 (68.9%) | 79 (66.9%) | 833 (49.9%) | 582 (40.8%) |  |
| Race/Ethnicity |  |  |  |  |  |  | **<0.001** |
| Mexican American | 574 (15.2%) | 18 (6.1%) | 10 (3.8%) | 16 (13.6%) | 187 (11.2%) | 343 (24.1%) |  |
| Other Hispanic | 375 (9.9%) | 33 (11.3%) | 22 (8.3%) | 5 (4.2%) | 199 (11.9%) | 116 (8.1%) |  |
| Non-Hispanic White | 1305 (34.6%) | 140 (47.8%) | 147 (55.7%) | 56 (47.5%) | 536 (32.1%) | 426 (29.9%) |  |
| Non-Hispanic Black | 994 (26.4%) | 79 (27%) | 34 (12.9%) | 35 (29.7%) | 405 (24.3%) | 441 (30.9%) |  |
| Other Race, including Multi-Racial | 522 (13.8%) | 23 (7.8%) | 51 (19.3%) | 6 (5.1%) | 342 (20.5%) | 100 (7%) |  |
| BMI (kg/m^2^) | 28.3 (24.7-32.9) | 27.7 (24.5-33.1) | 26.9 (23.5-31.1) | 29.6 (26.7-33.5) | 28 (24.6-32.5) | 28.8 (25.1-33.5) | **<0.001** |
| Income-poverty ratio | 1.8 (1.0-4.0) | 1.1 (0.8-2.0) | 3.7 (2.1-5.0) | 1.3 (0.8-3.1) | 2.6 (1.3-4.9) | 1.3 (0.8-2.6) | **<0.001** |
| Education level |  |  |  |  |  |  | **<0.001** |
| Less than 9th grade | 433 (11.5%) | 56 (19.1%) | 6 (2.3%) | 9 (7.6%) | 138 (8.3%) | 224 (15.7%) |  |
| 9-11th grade | 632 (16.8%) | 84 (28.7%) | 13 (4.9%) | 19 (16.1%) | 190 (11.4%) | 326 (22.9%) |  |
| High school grad/GED or Equivalent | 834 (22.1%) | 73 (24.9%) | 32 (12.1%) | 41 (34.7%) | 321 (19.2%) | 367 (25.7%) |  |
| Some college or AA degree | 1013 (26.9%) | 64 (21.8%) | 68 (25.8%) | 39 (33.1%) | 479 (28.7%) | 363 (25.5%) |  |
| College graduate or above | 856 (22.7%) | 16 (5.5%) | 145 (54.9%) | 10 (8.5%) | 540 (32.4%) | 145 (10.2%) |  |
| Missing | 2 (0.1%) | 0 (0.0%) | 0 (0.0%) | 0 (0.0%) | 1 (0.1%) | 1 (0.1%) |  |
| Alcohol consumption |  |  |  |  |  |  | **<0.001** |
| Never | 687 (18.2%) | 103 (35.2%) | 24 (9.1%) | 23 (19.5%) | 243 (14.6%) | 294 (20.6%) |  |
| Everyday/drink | 158 (4.2%) | 11 (3.8%) | 4 (1.5%) | 3 (2.5%) | 65 (3.9%) | 75 (5.3%) |  |
| Every week/drink | 930 (24.7%) | 41 (14.0%) | 92 (34.8%) | 19 (16.1%) | 431 (25.8%) | 347 (24.3%) |  |
| Every month/drink | 532 (14.1%) | 28 (9.6%) | 52 (19.7%) | 12 (10.2%) | 257 (15.4%) | 183 (12.8%) |  |
| Several month/drink | 677 (18.0%) | 57 (19.5%) | 43 (16.3%) | 36 (30.5%) | 312 (18.7%) | 229 (16.1%) |  |
| Missing | 786 (20.8%) | 53 (18.1%) | 49 (18.6%) | 25 (21.2%) | 361 (21.6%) | 298 (20.9%) |  |
| Smoking status |  |  |  |  |  |  | **<0.001** |
| Never | 1949 (51.7%) | 65 (22.2%) | 191 (72.3%) | 69 (58.5%) | 987 (59.1%) | 637 (44.7%) |  |
| Former smoker | 867 (23%) | 94 (32.1%) | 52 (19.7%) | 18 (15.3%) | 397 (23.8%) | 306 (21.5%) |  |
| <10/day | 365 (9.7%) | 36 (12.3%) | 11 (4.2%) | 12 (10.2%) | 131 (7.8%) | 175 (12.3%) |  |
| 10-20/day | 280 (7.4%) | 27 (9.2%) | 6 (2.3%) | 9 (7.6%) | 90 (5.4%) | 148 (10.4%) |  |
| ≥20/day | 309 (8.2%) | 71 (24.2%) | 4 (1.5%) | 10 (8.5%) | 64 (3.8%) | 160 (11.2%) |  |
| Hypertension status |  |  |  |  |  |  | **<0.001** |
| Normal | 1531 (40.6%) | 97 (33.1%) | 145 (54.9%) | 64 (54.2%) | 695 (41.6%) | 530 (37.2%) |  |
| Elevated | 614 (16.3%) | 48 (16.4%) | 44 (16.7%) | 10 (8.5%) | 272 (16.3%) | 240 (16.8%) |  |
| Hypertension stage 1 | 845 (22.4%) | 59 (20.1%) | 44 (16.7%) | 24 (20.3%) | 396 (23.7%) | 322 (22.6%) |  |
| Hypertension stage 2 | 634 (16.8%) | 77 (26.3%) | 21 (8%) | 15 (12.7%) | 249 (14.9%) | 272 (19.1%) |  |
| Hypertension crisis | 32 (0.8%) | 6 (2%) | 0 (0.0%) | 0 (0.0%) | 7 (0.4%) | 19 (1.3%) |  |
| Missing | 114 (3%) | 6 (2%) | 10 (3.8%) | 5 (4.2%) | 50 (3%) | 43 (3%) |  |
| Diabetes status |  |  |  |  |  |  | **<0.001** |
| Non-diabetes | 1986 (52.7%) | 102 (34.8%) | 187 (70.8%) | 77 (65.3%) | 922 (55.2%) | 698 (48.9%) |  |
| Prediabetes | 1139 (30.2%) | 99 (33.8%) | 60 (22.7%) | 31 (26.3%) | 494 (29.6%) | 455 (31.9%) |  |
| Diabetes | 286 (7.6%) | 37 (12.6%) | 8 (3.0%) | 5 (4.2%) | 115 (6.9%) | 121 (8.5%) |  |
| Poorly controlled diabetes | 200 (5.3%) | 30 (10.2%) | 3 (1.1%) | 0 (0.0%) | 77 (4.6%) | 90 (6.3%) |  |
| Missing | 159 (4.2%) | 25 (8.5%) | 6 (2.3%) | 5 (4.2%) | 61 (3.7%) | 62 (4.3%) |  |

*Note*:

Edentulous: individuals with no natural teeth present in oral cavity; Oral health: individuals with neither caries and nor periodontitis, as stage I periodontitis were considered as no periodontitis defined by ACES definition; Caries: individuals with caries present and with stage I periodontitis based on ACES definition; Periodontitis: individuals with no caries and with stage II-IV periodontitis based on ACES definition; Caries+Periodontitis: individuals with both caries present and with stage II-IV periodontitis based on ACES definition.

BMI (body mass index): calculated as weight (kg) divided by height squared (m^2^).

Income-poverty ratio: calculated by dividing total family income by the poverty threshold based on poverty guidelines, specific to family size, year and state.

Hypertension status: categorized based on examined systolic and diastolic blood pressure values (SBP and DBP). Normal: SBP <120 mmHg and DBP <80 mmHg; Elevated: SBP 120-129 mmHg and DBP <80 mmHg; hypertension stage I: SBP 130-139 mmHg or DBP 80-89 mmHg; hypertension stage II: SBP ≥140 mmHg or DBP ≥90 mmHg; hypertension crisis: SBP >180 mmHg and/or DBP >120 mmHg.

Diabetes status: defined by tested glycohemoglobin (HbA1c) levels. Non-diabetes: HbA1c <5.7%; Prediabetes: HbA1c 5.7-6.4%; Diabetes: HbA1c ≥6.5%; Poorly controlled diabetes: HbA1c ≥8.0%.

**Table S2 Characteristics of study participants without caries and with different status of periodontitis according to CDC/AAP periodontitis definitions.**

| **CDC/AAP definition** | **Overall** | **No Periodontitis** | **Mild Periodontitis** | **Moderate Periodontitis** | **Severe Periodontitis** | ***p* value** |
| --- | --- | --- | --- | --- | --- | --- |
| **N** | 1933 | 1056 | 72 | 592 | 213 |  |
| Age | 48 (39-58) | 44 (36-54) | 43 (37-52) | 53 (44-62) | 54 (46-62) | **<0.001** |
| Sex |  |  |  |  |  | **<0.001** |
| Male | 918 (47.5%) | 417 (39.5%) | 43 (59.7%) | 300 (50.7%) | 158 (74.2%) |  |
| Female | 1015 (52.5%) | 639 (60.5%) | 29 (40.3%) | 292 (49.3%) | 55 (25.8%) |  |
| Race/Ethnicity |  |  |  |  |  | **<0.001** |
| Mexican American | 197 (10.2%) | 88 (8.3%) | 8 (11.1%) | 69 (11.7%) | 32 (15%) |  |
| Other Hispanic | 221 (11.4%) | 111 (10.5%) | 12 (16.7%) | 74 (12.5%) | 24 (11.3%) |  |
| Non-Hispanic White | 683 (35.3%) | 458 (43.4%) | 27 (37.5%) | 158 (26.7%) | 40 (18.8%) |  |
| Non-Hispanic Black | 439 (22.7%) | 188 (17.8%) | 11 (15.3%) | 165 (27.9%) | 75 (35.2%) |  |
| Other Race, including Multi-Racial | 393 (20.3%) | 211 (20%) | 14 (19.4%) | 126 (21.3%) | 42 (19.7%) |  |
| BMI (kg/m^2^) | 27.8 (24.5-32.3) | 27.3 (24-31.8) | 28.8 (25.9-34.1) | 28.8 (25-33.2) | 27.7 (24.4-31.7) | **<0.001** |
| Income-poverty ratio | 2.8 (1.3-5.0) | 3.6 (1.6-5.0) | 2.2 (1.3-4.3) | 2.2 (1.2-4.4) | 1.7 (1.1-3.2) | **<0.001** |
| Education level |  |  |  |  |  |  |
| Less than 9th grade | 144 (7.4%) | 46 (4.4%) | 5 (6.9%) | 62 (10.5%) | 31 (14.6%) | **<0.001** |
| 9-11th grade | 203 (10.5%) | 86 (8.1%) | 3 (4.2%) | 83 (14.0%) | 31 (14.6%) |  |
| High school grad/GED or Equivalent | 353 (18.3%) | 140 (13.3%) | 18 (25.0%) | 136 (23%) | 59 (27.7%) |  |
| Some college or AA degree | 547 (28.3%) | 294 (27.8%) | 23 (31.9%) | 169 (28.5%) | 61 (28.6%) |  |
| College graduate or above | 685 (35.4%) | 490 (46.4%) | 23 (31.9%) | 141 (23.8%) | 31 (14.6%) |  |
| Missing | 1 (0.1%) | 0 (0.0%) | 0 (0.0%) | 1 (0.2%) | 0 (0.0%) |  |
| Alcohol consumption |  |  |  |  | <0.001 |  |
| Never | 267 (13.8%) | 112 (10.6%) | 6 (8.3%) | 109 (18.4%) | 40 (18.8%) | **<0.001** |
| Everyday/drink | 69 (3.6%) | 25 (2.4%) | 2 (2.8%) | 26 (4.4%) | 16 (7.5%) |  |
| Every week/drink | 523 (27.1%) | 309 (29.3%) | 20 (27.8%) | 126 (21.3%) | 68 (31.9%) |  |
| Every month/drink | 309 (16.0%) | 192 (18.2%) | 12 (16.7%) | 79 (13.3%) | 26 (12.2%) |  |
| Several month/drink | 355 (18.4%) | 193 (18.3%) | 17 (23.6%) | 117 (19.8%) | 28 (13.1%) |  |
| Missing | 410 (21.2%) | 225 (21.3%) | 15 (20.8%) | 135 (22.8%) | 35 (16.4%) |  |
| Smoking status |  |  |  |  | <0.001 |  |
| Never | 1178 (60.9%) | 732 (69.3%) | 53 (73.6%) | 312 (52.7%) | 81 (38.0%) | **<0.001** |
| Former smoker | 449 (23.2%) | 225 (21.3%) | 10 (13.9%) | 149 (25.2%) | 65 (30.5%) |  |
| <10/day | 142 (7.3%) | 51 (4.8%) | 3 (4.2%) | 57 (9.6%) | 31 (14.6%) |  |
| 10-20/day | 96 (5.0%) | 27 (2.6%) | 4 (5.6%) | 45 (7.6%) | 20 (9.4%) |  |
| ≥20/day | 68 (3.5%) | 21 (2.0%) | 2 (2.8%) | 29 (4.9%) | 16 (7.5%) |  |
| Hypertension status |  |  |  |  | <0.001 |  |
| Normal | 840 (43.5%) | 533 (50.5%) | 30 (41.7%) | 208 (35.1%) | 69 (32.4%) | **<0.001** |
| Elevated | 316 (16.3%) | 153 (14.5%) | 13 (18.1%) | 109 (18.4%) | 41 (19.2%) |  |
| Hypertension stage 1 | 440 (22.8%) | 219 (20.7%) | 17 (23.6%) | 159 (26.9%) | 45 (21.1%) |  |
| Hypertension stage 2 | 270 (14.0%) | 117 (11.1%) | 9 (12.5%) | 94 (15.9%) | 50 (23.5%) |  |
| Hypertension crisis | 7 (0.4%) | 1 (0.1%) | 0 (0.0%) | 3 (0.5%) | 3 (1.4%) |  |
| Missing | 60 (3.1%) | 33 (3.1%) | 3 (4.2%) | 19 (3.2%) | 5 (2.3%) |  |
| Diabetes status |  |  |  |  |  |  |
| Non-diabetes | 1109 (57.4%) | 702 (66.5%) | 46 (63.9%) | 269 (45.4%) | 92 (43.2%) | **<0.001** |
| Prediabetes | 554 (28.7%) | 244 (23.1%) | 18 (25.0%) | 218 (36.8%) | 74 (34.7%) |  |
| Diabetes | 123 (6.4%) | 52 (4.9%) | 5 (6.9%) | 46 (7.8%) | 20 (9.4%) |  |
| Poorly controlled diabetes | 80 (4.1%) | 22 (2.1%) | 2 (2.8%) | 36 (6.1%) | 20 (9.4%) |  |
| Missing | 67 (3.5%) | 36 (3.4%) | 1 (1.4%) | 23 (3.9%) | 7 (3.3%) |  |

*Note*:

Under the CDC/AAP definition, no periodontitis: individual absence of mild, moderate, or severe periodontitis found; Mild periodontitis: individual with ≥2 interproximal sites with attachment loss ≥3 mm, and ≥2 interproximal sites with pocket depth ≥4 mm (not on same tooth) or one site with pocket depth ≥5 mm; Moderate periodontitis: individual with ≥2 interproximal sites with attachment loss ≥4 mm (not on same tooth), or ≥2 interproximal sites with pocket depth ≥5 mm (not on same tooth); Severe periodontitis: individual with ≥2 interproximal sites with attachment loss ≥6 mm (not on same tooth) and ≥1 interproximal site with pocket depth ≥5 mm.

BMI (body mass index): calculated as weight (kg) divided by height squared (m^2^).

Income-poverty ratio: calculated by dividing total family income by the poverty threshold based on poverty guidelines, specific to family size, year and state.

Hypertension status: categorized based on examined systolic and diastolic blood pressure values (SBP and DBP). Normal: SBP <120 mmHg and DBP <80 mmHg; Elevated: SBP 120-129 mmHg and DBP <80 mmHg; hypertension stage I: SBP 130-139 mmHg or DBP 80-89 mmHg; hypertension stage II: SBP ≥140 mmHg or DBP ≥90 mmHg; hypertension crisis: SBP >180 mmHg and/or DBP >120 mmHg.

Diabetes status: defined by tested glycohemoglobin (HbA1c) levels. Non-diabetes: HbA1c <5.7%; Prediabetes: HbA1c 5.7-6.4%; Diabetes: HbA1c ≥6.5%; Poorly controlled diabetes: HbA1c ≥8.0%.

**Table S3 Characteristics of study participants without caries and with different status of periodontitis according to ACES periodontitis definitions.**

| **ACES definition** | **Overall** | **Stage I Periodontitis** | **Stage II Periodontitis** | **Stage III/IV Periodontitis** | ***p* value** |
| --- | --- | --- | --- | --- | --- |
| **N** | 1933 | 264 | 1000 | 669 |  |
| Age | 48 (39-58) | 40 (33-48) | 46 (37-56) | 55 (45-62) | **<0.001** |
| Sex |  |  |  |  | **<0.001** |
| Male | 918 (47.5%) | 82 (31.1%) | 445 (44.5%) | 391 (58.4%) |  |
| Female | 1015 (52.5%) | 182 (68.9%) | 555 (55.5%) | 278 (41.6%) |  |
| Race/Ethnicity |  |  |  |  | **<0.001** |
| Mexican American | 197 (10.2%) | 10 (3.8%) | 107 (10.7%) | 80 (12%) |  |
| Other Hispanic | 221 (11.4%) | 22 (8.3%) | 114 (11.4%) | 85 (12.7%) |  |
| Non-Hispanic White | 683 (35.3%) | 147 (55.7%) | 367 (36.7%) | 169 (25.3%) |  |
| Non-Hispanic Black | 439 (22.7%) | 34 (12.9%) | 203 (20.3%) | 202 (30.2%) |  |
| Other Race, including Multi-Racial | 393 (20.3%) | 51 (19.3%) | 209 (20.9%) | 133 (19.9%) |  |
| BMI (kg/m^2^) | 27.8 (24.5-32.3) | 26.9 (23.5-31.1) | 27.8 (24.5-32.1) | 28.2 (25.0-32.8) | **<0.001** |
| Income-poverty ratio | 2.8 (1.3-5.0) | 3.7 (2.1-5.0) | 3.1 (1.4-5.0) | 2.0 (1.1-4.0) | **<0.001** |
| Education level |  |  |  |  | **<0.001** |
| Less than 9th grade | 144 (7.4%) | 6 (2.3%) | 57 (5.7%) | 81 (12.1%) |  |
| 9-11th grade | 203 (10.5%) | 13 (4.9%) | 92 (9.2%) | 98 (14.6%) |  |
| High school grad/GED or Equivalent | 353 (18.3%) | 32 (12.1%) | 146 (14.6%) | 175 (26.2%) |  |
| Some college or AA degree | 547 (28.3%) | 68 (25.8%) | 295 (29.5%) | 184 (27.5%) |  |
| College graduate or above | 685 (35.4%) | 145 (54.9%) | 410 (41.0%) | 130 (19.4%) |  |
| Missing | 1 (0.1%) | 0 (0.0%) | 0 (0.0%) | 1 (0.1%) |  |
| Alcohol consumption |  |  |  |  | **<0.001** |
| Never | 267 (13.8%) | 24 (9.1%) | 115 (11.5%) | 128 (19.1%) |  |
| Everyday/drink | 69 (3.6%) | 4 (1.5%) | 28 (2.8%) | 37 (5.5%) |  |
| Every week/drink | 523 (27.1%) | 92 (34.8%) | 264 (26.4%) | 167 (25.0%) |  |
| Every month/drink | 309 (16.0%) | 52 (19.7%) | 174 (17.4%) | 83 (12.4%) |  |
| Several month/drink | 355 (18.4%) | 43 (16.3%) | 187 (18.7%) | 125 (18.7%) |  |
| Missing | 410 (21.2%) | 49 (18.6%) | 232 (23.2%) | 129 (19.3%) |  |
| Smoking status |  |  |  |  | **<0.001** |
| Never | 1178 (60.9%) | 191 (72.3%) | 673 (67.3%) | 314 (46.9%) |  |
| Former smoker | 449 (23.2%) | 52 (19.7%) | 219 (21.9%) | 178 (26.6%) |  |
| <10/day | 142 (7.3%) | 11 (4.2%) | 54 (5.4%) | 77 (11.5%) |  |
| 10-20/day | 96 (5.0%) | 6 (2.3%) | 33 (3.3%) | 57 (8.5%) |  |
| ≥20/day | 68 (3.5%) | 4 (1.5%) | 21 (2.1%) | 43 (6.4%) |  |
| Hypertension status |  |  |  |  | **<0.001** |
| Normal | 840 (43.5%) | 145 (54.9%) | 476 (47.6%) | 219 (32.7%) |  |
| Elevated | 316 (16.3%) | 44 (16.7%) | 139 (13.9%) | 133 (19.9%) |  |
| Hypertension stage 1 | 440 (22.8%) | 44 (16.7%) | 227 (22.7%) | 169 (25.3%) |  |
| Hypertension stage 2 | 270 (14.0%) | 21 (8.0%) | 123 (12.3%) | 126 (18.8%) |  |
| Hypertension crisis | 7 (0.4%) | 0 (0.0%) | 1 (0.1%) | 6 (0.9%) |  |
| Missing | 60 (3.1%) | 10 (3.8%) | 34 (3.4%) | 16 (2.4%) |  |
| Diabetes status |  |  |  |  | **<0.001** |
| Non-diabetes | 1109 (57.4%) | 187 (70.8%) | 625 (62.5%) | 297 (44.4%) |  |
| Prediabetes | 554 (28.7%) | 60 (22.7%) | 258 (25.8%) | 236 (35.3%) |  |
| Diabetes | 123 (6.4%) | 8 (3.0%) | 56 (5.6%) | 59 (8.8%) |  |
| Poorly controlled diabetes | 80 (4.1%) | 3 (1.1%) | 25 (2.5%) | 52 (7.8%) |  |
| Missing | 67 (3.5%) | 6 (2.3%) | 36 (3.6%) | 25 (3.7%) |  |

*Note*:

Under the ACES definition, stage I periodontitis: individual with interproximal attachment loss ≥1 mm at ≥2 non-adjacent teeth, with maximum attachment loss of 1-2 mm; Stage II periodontitis: individual with interproximal attachment loss ≥1 mm at ≥2 non-adjacent teeth, with maximum attachment loss of 3-4 mm; Stage III/IV periodontitis: individual with interproximal attachment loss ≥1 mm at ≥2 non-adjacent teeth, with maximum attachment loss ≥5 mm.

BMI (body mass index): calculated as weight (kg) divided by height squared (m^2^).

Income-poverty ratio: calculated by dividing total family income by the poverty threshold based on poverty guidelines, specific to family size, year and state.

Hypertension status: categorized based on examined systolic and diastolic blood pressure values (SBP and DBP). Normal: SBP <120 mmHg and DBP <80 mmHg; Elevated: SBP 120-129 mmHg and DBP <80 mmHg; hypertension stage I: SBP 130-139 mmHg or DBP 80-89 mmHg; hypertension stage II: SBP ≥140 mmHg or DBP ≥90 mmHg; hypertension crisis: SBP >180 mmHg and/or DBP >120 mmHg.

Diabetes status: defined by tested glycohemoglobin (HbA1c) levels. Non-diabetes: HbA1c <5.7%; Prediabetes: HbA1c 5.7-6.4%; Diabetes: HbA1c ≥6.5%; Poorly controlled diabetes: HbA1c ≥8.0%.

**Table S4 Characteristics of the local cohort participants in external validation with different status of periodontitis according to the CDC/AAP periodontitis definitions.**

| **Variables** | **All (n=392)** | **No Periodontitis  (n=60)** | **Mild Periodontitis  (n=3)** | **Moderate Periodontitis  (n=279)** | **Severe Periodontitis  (n=50)** | ***p* value** |
| --- | --- | --- | --- | --- | --- | --- |
| **Age** | 24.0 (21.0–34.0) | 25.0 (22.0–32.0) | 25.0 (24.0–33.0) | 23.0 (21.0–28.0) | 48.0 (42.0–57.0) | **<0.001** |
| **Sex** |  |  |  |  |  | **<0.001** |
| Male | 132 (33.7%) | 9 (15%) | 1 (33.3%) | 90 (32.3%) | 32 (64%) |  |
| Female | 260 (66.3%) | 51 (85%) | 2 (66.7%) | 189 (67.7%) | 18 (36%) |  |
| **Race** |  |  |  |  |  | **<0.001** |
| Han Chinese | 353 (90.1%) | 51 (85%) | 3 (100%) | 250 (89.6%) | 49 (98%) |  |
| Chinese ethnic minority groups | 32 (8.2%) | 3 (5%) | 0 (0%) | 28 (10%) | 1 (2%) |  |
| White | 7 (1.8%) | 6 (10%) | 0 (0%) | 1 (0.4%) | 0 (0%) |  |
| **Education Level** |  |  |  |  |  | **<0.001** |
| No Education/Elementary School/Junior High School | 9 (2.3%) | 0 (0%) | 0 (0%) | 4 (1.4%) | 5 (10%) |  |
| Senior high school/Technical secondary school | 21 (5.4%) | 2 (3.3%) | 0 (0%) | 14 (5%) | 5 (10%) |  |
| Junior college/Bachelor | 236 (60.2%) | 26 (43.3%) | 1 (33.3%) | 173 (62%) | 36 (72%) |  |
| Master/Doctor | 124 (31.6%) | 31 (51.7%) | 2 (66.7%) | 87 (31.2%) | 4 (8%) |  |
| Missing | 2 (0.5%) | 1 (1.7%) | 0 (0%) | 1 (0.4%) | 0 (0%) |  |
| **Family income** |  |  |  |  |  | 0.276 |
| Low income | 82 (20.9%) | 10 (16.7%) | 1 (33.3%) | 63 (22.6%) | 8 (16%) |  |
| Medium income | 203 (51.8%) | 25 (41.7%) | 1 (33.3%) | 150 (53.8%) | 27 (54%) |  |
| High income | 72 (18.4%) | 18 (30%) | 1 (33.3%) | 42 (15.1%) | 11 (22%) |  |
| Don't know | 35 (8.9%) | 7 (11.7%) | 0 (0%) | 24 (8.6%) | 4 (8%) |  |
| **Tooth numbers** | 28.0 (26.0–28.0) | 28.0 (27.0–28.0) | 25.0 (24.0–25.0) | 28.0 (27.0–28.0) | 26.0 (24.0–27.0) | **<0.001** |
| **Alcohol consumption** |  |  |  |  |  | **0.005** |
| No | 152 (38.8%) | 25 (41.7%) | 1 (33.3%) | 114 (40.9%) | 12 (24%) |  |
| Yes | 233 (59.4%) | 33 (55%) | 2 (66.7%) | 163 (58.4%) | 35 (70%) |  |
| Former consumption | 4 (1%) | 0 (0%) | 0 (0%) | 1 (0.4%) | 3 (6%) |  |
| Missing | 3 (0.8%) | 2 (3.3%) | 0 (0%) | 1 (0.4%) | 0 (0%) |  |
| **Smoking status** |  |  |  |  |  | **<0.001** |
| Never | 352 (89.8%) | 58 (96.7%) | 3 (100%) | 258 (92.5%) | 33 (66%) |  |
| Former smoker | 17 (4.3%) | 1 (1.7%) | 0 (0%) | 7 (2.5%) | 9 (18%) |  |
| Current smoker | 13 (3.3%) | 1 (1.7%) | 0 (0%) | 9 (3.2%) | 3 (6%) |  |
| Heavy smoker | 10 (2.6%) | 0 (0%) | 0 (0%) | 5 (1.8%) | 5 (10%) |  |
| **Diabetes status**^†^ |  |  |  |  |  | **0.003** |
| No | 390 (99.5%) | 60 (100%) | 3 (100%) | 279 (100%) | 48 (96%) |  |
| Yes | 2 (0.5%) | 0 (0%) | 0 (0%) | 0 (0%) | 2 (4%) |  |
| **Cardiovascular disease status** |  |  |  |  |  | 0.097 |
| No | 377 (96.2%) | 59 (98.3%) | 3 (100%) | 270 (96.8%) | 45 (90%) |  |
| Yes | 15 (3.8%) | 1 (1.7%) | 0 (0%) | 9 (3.2%) | 5 (10%) |  |

*Note*:

For family income, low income means 0–5000 Renminbi (RMB, Chinese Yuan) per person per month, medium income means 5000–15000 RMB, and high income means >15000 RMB. Conversion rate: 1 RMB≈0.14 USD (December 2025).

Heavy smoker means smoking more than 10 cigarettes per day.

For alcohol consumption, “No” refers to individuals who have never consumed alcoholic beverages, “Yes” refers to those who currently consume alcoholic beverages (regardless of frequency or quantity), and “Former consumption” refers to individuals who previously consumed alcohol but no longer do so.

Diabetes status and cardiovascular disease status was recorded based on participants’ self-reporting.

**See in attached files:**

Supplementary Spreadsheet 1-differential abundance by ALDEx2 in different oral conditions

Supplementary Spreadsheet 2-differential abundance by DESeq2 in different oral conditions

Supplementary Spreadsheet 3- MaAsLin2 results for linear associations between CLR-transformed genus-level data and oral conditions defined by caries status and the ACES periodontitis classification, under multiple covariate-adjusted models

Supplementary Spreadsheet 4-Taxa identified in the Venn comparison of ALDEx2 and DESeq2 differential abundance analyses under identical oral condition contrasts

Supplementary Spreadsheet 5-Taxa identified in the Venn comparison of ALDEx2 and DESeq2 differential abundance analyses under identical periodontitis status contrasts

1. & Online SMDI platform: <https://bioinformatics.forsyth.org/smdi/index.php> [↑](#footnote-ref-1)
